# Neural Mechanisms of Bidirectional Visuo‐Linguistic Transformation in Interactive Communication

**DOI:** 10.1002/hbm.70540

**Published:** 2026-05-12

**Authors:** Yulei Shen, Takahiko Koike, Shohei Tsuchimoto, Ayumi Yoshioka, Kanae Ogasawara, Norihiro Sadato, Hiroki C. Tanabe

**Affiliations:** ^1^ Department of Cognitive & Psychological Sciences, Graduate School of Informatics Nagoya University Nagoya Japan; ^2^ Inter‐Brain Dynamics Collaboration Unit, RIKEN Center for Brain Science Wako Japan; ^3^ Section of Brain Function Information National Institute for Physiological Sciences (NIPS) Okazaki Japan; ^4^ Division of Neural Dynamics NIPS Okazaki Japan; ^5^ Research Organization of Science and Technology Ritsumeikan University Kusatsu Japan

**Keywords:** dynamic causal model, hyperscan fMRI, online interaction, semantic control, speech, verbal communication, visual imagery

## Abstract

Human communication requires the flexible transformation of visual input into verbal descriptions and the reconstruction of mental imagery from language. However, the neural mechanisms underlying these bidirectional transformations during social interaction remain poorly understood. Using hyperscanning fMRI and a role‐switching communicative task, we investigated how individuals alternately encode visual stimuli into language and decode language into mental imagery within an interactive context. Whole‐brain analyses identified brain regions implicated in these processes. Based on these findings, we selected three key regions—the inferior frontal gyrus (IFG), fusiform face area (FFA), and intraparietal sulcus (IPS)—and applied dynamic causal modeling (DCM) to examine their effective connectivity. The DCM results revealed that connectivity among IFG, FFA, and IPS dynamically reconfigures to support role‐dependent shifts between top‐down and bottom‐up information flow. These findings highlight a flexible, coordinated neural architecture that integrates visual and linguistic information, offering new insights into how communication arises from the interplay between perceptual and language systems.

## Graphical abstract

Using hyperscanning fMRI and a role‐switching task, we examined how individuals transform visual input into language and reconstruct imagery from speech. Whole‐brain analyses identified key regions, and effective connectivity among IFG, FFA, and IPS revealed dynamic, role‐dependent shifts in information flow direction.

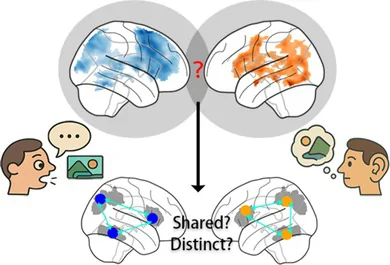

## 1 Introduction

Human communication depends on flexible visuo‐linguistic transformation: the bidirectional conversion between integrated visual percepts and discrete linguistic codes (Barsalou 2008; Pulvermüller 2013). In everyday interactions, individuals frequently alternate between describing what they see and imagining what others describe. When a visually rich stimulus must be verbally conveyed, people select and articulate salient perceptual features—a process that can be understood as verbalization. Conversely, when interpreting verbal descriptions, people reconstruct a visual mental model from linguistic cues—a process of visualization. These complementary transformations form the basis for what we refer to as communicative roles. In the sender role, individuals describe visual features to a partner; in the receiver role, they generate mental imagery based on those descriptions. Understanding the neural mechanisms that support flexible switching between these roles is essential for characterizing interactive communication.

Neuroimaging research on language production and visual imagery has shown that verbalization and visualization recruit distributed but partly overlapping neural systems with opposite dominant directions of information flow. Verbalizing visual content typically involves a processing cascade initiated by perceptual input in visual regions and progressing through temporal semantic areas to frontal language‐output regions (Fedorenko and Blank 2020; Matchin and Hickok 2020; Hagoort 2014; Indefrey and Levelt 2004). In contrast, the generation of visual imagery is driven by endogenous signals originating from frontal executive and parietal attention systems, which reconstruct perceptual representations in visual cortex in the absence of external input (Dijkstra et al. 2017, 2019; Pearson 2019; Winlove et al. 2018; Fulford et al. 2018). However, because previous studies have examined these processes in isolation and outside of communicative contexts, it remains unclear how the brain shifts flexibly between these opposing modes during rapid alternation of information sender and receiver roles.

This flexibility likely relies on regulatory architecture capable of dynamically coordinating semantic and perceptual systems. Research on semantic control identifies a suitable candidate: the Semantic Control Network (SCN), comprising the left inferior frontal gyrus (IFG), posterior middle temporal gyrus (pMTG), posterior inferior temporal gyrus (pITG), and inferior parietal cortex (IPL/IPS) (Ralph et al. 2017; Wang et al. 2024). Within this network, the IFG supports controlled semantic retrieval; the pMTG/pITG integrate semantic and perceptual information, serving as intermediaries between control regions and category‐selective sensory cortices (Jackson 2021); and parietal components contribute to spatial processing and attentional allocation relevant to both visual encoding and imagery (Dijkstra et al. 2019; Pearson 2019; Spagna et al. 2021). These properties suggest that both communicative roles may rely on a shared SCN‐anchored network coupled with modality‐specific visual regions, but with role‐dependent differences in the direction of information flow.

To investigate this possibility in a naturalistic communicative setting, we used a hyperscanning fMRI paradigm (Jiang et al. 2012; Redcay and Schilbach 2019) that allowed two participants to alternate between describing facial features and reconstructing mental images of faces during real‐time interaction. This design enabled direct comparison of the opposing visuo‐linguistic transformations within matched interpersonal contexts, and the analyses focused on two complementary aspects: the regional architecture of the network and the directionality of effective connectivity within it. We assumed that both communicative roles would engage a shared network including SCN regions (IFG, pMTG/pITG, IPL/IPS) and category‐selective visual areas, especially the fusiform face area (FFA) given use of facial stimuli. At the connectivity level, we examined whether the direction of information flow within this network varies as a function of communicative role. Visualization was hypothesized to be characterized by dominant top‐down connectivity from semantic control regions to visual cortex, reflecting the internally driven reconstruction of visual representations. In contrast, verbalization was expected to involve a more distributed connectivity profile encompassing both bottom‐up and top‐down pathways, as facial description requires concurrent perceptual analysis, semantic control, and lexical selection. Because such directional predictions do not uniquely specify a single network configuration, we adopted an exploratory approach to identify the connectivity structure best supported by the data. Furthermore, by manipulating the richness of descriptive information, we investigated how regional activation and effective connectivity are modulated under sparse versus rich information conditions.

## 2 Method

### 2.1 Participants

Forty‐six participants (mean age: 26.3 years, standard deviation (SD): 7.26, age range: 19–43) were paired (including 5 male–male pairs and 18 female–female pairs) to participate in the study. Prior to the experiment, these individuals did not know their pair partner. All participants were right‐handed and had no reported history of neurological or psychiatric disorders, nor any history of head injury. Their right‐handedness was confirmed using the Edinburgh Handedness Inventory (Oldfield 1971). The study protocol was approved by the ethics committee of Nagoya University, Nagoya, Japan and the ethics committee of the National Institute for Physiological Sciences (NIPS), Okazaki, Japan. Participants provided written informed consent before the experiment.

### 2.2 Experiment Procedure

#### 2.2.1 Hyperscan MRI Setup

Two MRI scanners were strategically installed side by side, with their magnetic fields aligned in parallel to minimize interference. The scanners were connected to a common control room, and the scan initiation was precisely synchronized using an external trigger generated by custom‐built MS‐DOS software. To realize real‐time interactions between pairs of participants, the MRI scanners were connected to each other via microphones and noise‐cancelling headphones (Opto ACTIVE II, Kobatel, Yokohama, Japan), so that participants could seamlessly communicate verbally during the MR data acquisition. The visual stimuli presented during the formal scanning task were presented using the Presentation software package (Neurobehavioral Systems, Albany, CA, USA) (RRID: SCR_002521).

#### 2.2.2 Task

**FIGURE 1:**
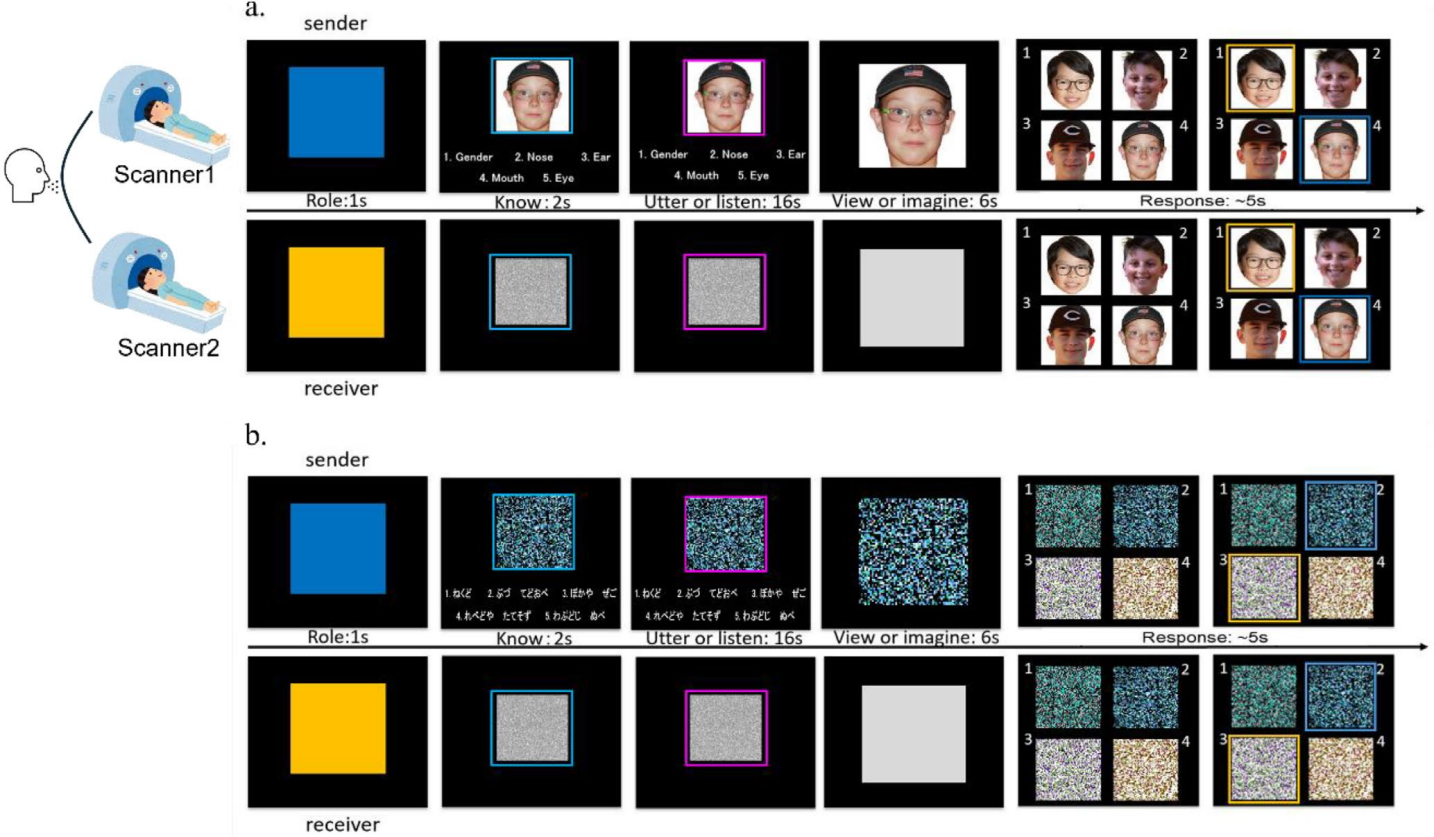
Experimental procedure of one trial. (a) Face condition. (b) Control condition.

We designed a novel introduction‐response hyperscan task (Fig. 1) to simulate natural conversation in a semi‐constrained setting. Each pair of participants was randomly assigned to scanner 1 or 2 and engaged in real‐time dialogue to complete the task. Each trial comprised five phases. At the start, one participant (the sender) viewed a target picture and read corresponding hint cues (knowing period). Based on these cues, they generated sentences to introduce the picture to their partner (the receiver). Building on previous findings that providing more features of information enhances mental imagery vividness (Ganis et al. 2004; Cui et al. 2007) and that highly detailed descriptive language facilitates comprehension and emotional engagement (Paivio 1990, 2014; Djikic and Oatley 2014), we systematically manipulated information amount by varying the number of facial feature hints (2 vs. 5), requiring the sender to generate 2 or 5 sentences accordingly. During the utter‐listen period (16 s), the receiver responded verbally after each sentence to indicate comprehension. The timing of this phase was validated in our prior behavioral study (*N* = 22) to ensure that participants could complete both the 2‐hint and 5‐hint descriptions naturally and without time pressure. In the subsequent view‐imagery period (6 s), the receiver imagined the target image based on the sender's description, while the sender viewed the original picture. The duration was determined based on previous work on visual perception and visual mental imagery across unimodal and multimodal contexts (Bainbridge et al. 2021; de Borst et al. 2012; de Borst and de Gelder 2019; Herholz et al. 2012). At the end of each trial, both participants completed a four‐alternative forced‐choice (4AFC) task. The receiver selected the image that best matched the mental image constructed from the description, while the sender identified the original target image. The sender's response thus provided feedback for the receiver. To maintain response independence, both choices were revealed simultaneously via color‐coded frames only after both partners had submitted their answers. Participants alternated roles in each trial, with their assigned role indicated at the beginning of each trial (cue period). The stimuli included face‐only portraits and scrambled mosaic control images. In face trials (Figure 1a), hint cues referred to human facial features (e.g., “eye,” “mouth,” “hair”), allowing the sender to construct introductions constrained by the sentence structure “XX is …” (“XXは……です”). In control trials (Figure 1b), scrambled mosaic images were paired with randomly generated Hiragana nonsense syllables as hint cues. Senders used these meaningless words to form sentences, ensuring the condition lacked meaningful semantic content. To minimize inter‐dyad variance, all pairs followed the same trial sequence with identical stimuli and hint cues. The control condition was designed to rule out confounding effects of primary acoustic features and sentence structure, allowing us to isolate the neural mechanisms underlying higher‐level semantic and conceptual processing during conversation.

#### 2.2.3 Procedure

Prior to the start of the experiment, each pair of participants received instructions together outside the scanner and engaged in behavioral task practice. Once inside the scanner, participants were first asked to give a brief self‐introduction and then proceeded to a practice run with scanning. The practice run had the same number of trials as the formal task, but all the stimulus images used in the practice runs were not repeated in the formal runs. During the practice run, participants were reminded and corrected if they did not perform according to the experimental requirements (e.g., talking too long, not giving verbal responses to their partner, not following the prescribed sentence structure for introductions, or exceeding the time limit for making selections). This ensured that participants fully understood the task before beginning the formal task. The formal task consisted of 6 runs, with each run containing 16 trials and two trials for each condition (role × category × amount). Following the scan and a debriefing session, participants were asked to complete the Vividness of Visual Imagery Questionnaire (VVIQ) (Marks 1973) and the Verbalizer‐Visualizer Questionnaire (VVQ) (Richardson 1977) in Japanese. Participants also completed a post‐scan auditory working‐memory task consisting of 30 trials in which they recalled the order of five spoken words on each trial. This assessment was used to identify individuals with atypical working‐memory performance.

### 2.3 Stimulus

All images were face‐only portraits selected from the Flickr‐Faces‐HQ dataset (Karras et al. 2019) at a resolution of 600 × 600 pixels. The non‐head regions in the images were removed and replaced with a white background. Among them, 48 images served as target stimuli, while an additional 144 images were selected as 4AFC options. The selection criterion for the 4AFC option images required that each image shared at least one facial feature with the corresponding target image.

For the control condition, mosaic images were created by scrambling the face images (both target and 4AFC option images) into a mosaic pattern. This was done using the Photoshop Telegraphics Scramble filter with a scramble size of 10 pixels, followed by adjustments to the color levels.

### 2.4 MRI Acquisition

MRI time‐series data were acquired using two MRI scanners (Magnetom Verio 3 Tesla, Siemens, Erlangen, Germany) at NIPS, with standard 32‐channel phased array coils. Functional images were acquired using T2*‐weighted, gradient‐echoplanar imaging with the multi‐band sequence (TR = 1000 msec, TE = 31 msec, flip angle = 55°, multiband acceleration factor = 8), with each volume consisting of 72 slices of 2 mm thickness with 0 mm gap, 2 × 2 mm^2^ in‐plane resolution (field of view = 208 × 208 mm^2^). There are six functional scan runs of 16 trials each. Anatomical images were acquired using a T1‐weighted magnetization‐prepared rapid gradient‐echo (MP‐RAGE) sequence (TR = 2400 ms, TI = 1060 ms, TE = 2.24 ms, flip angle = 8°, 224 slices, 0.8 × 0.8 × 0.8 mm^3^ resolution, field of view = 256 × 240 mm^2^).

### 2.5 fMRI Preprocessing

Preprocessing was performed using AFNI (Cox 1996; Cox and Hyde 1997), FSL 6.0.7 (Jenkinson et al. 2012), and custom MATLAB R2021b scripts. Functional images were first corrected for distortion using FSL's topup and for transient intensity spikes using AFNI's 3dDespike. Head motion correction was then carried out with FSL's MCFLIRT. Next, we computed the transformations required for spatial normalization, including functional‐to‐structural co‐registration using Boundary‐Based Registration (BBR, 12 degrees of freedom) and nonlinear structural‐to‐MNI152 registration (10 mm warp resolution).

After motion correction, functional data from all six runs were temporally concatenated and submitted to multi‐run MELODIC ICA (Griffanti et al. 2014). Components were automatically evaluated using motion estimates and spatial masks from the earlier registration steps. Components were classified as noise if they showed high spatial overlap with noise‐related anatomical regions (e.g., ventricles > 20%, white matter > 95%, CSF > 30%), low‐frequency dominance (< 20% of power below 0.1 Hz), or strong correlation with the CSF signal (*r* > 0.3). These noise components were removed from the native‐space data using regression. The denoised functional images were then normalized to MNI152 space by applying the pre‐computed transformations and spatially smoothed with a 6‐mm FWHM Gaussian kernel.

### 2.6 Univariate Analysis

#### 2.6.1 First Level GLM Model

Activation analysis was performed using SPM12 (v7771). We specified two separate first‐level general linear models (GLMs)—one for the view–imagery period (GLM_vi) and one for the utter–listen period (GLM_ul)—to ensure orthogonality among the regressors of interest within each period. Each model included eight regressors of interest (role × category × amount), modeled at event onset. Regressor durations were set to 6 s for the view–imagery period and to 10 s (low amount) or 15 s (high amount) for the utter–listen period. Nuisance regressors included run‐specific constants and separate regressors (one per role and period) for the cue period, response period, and the task period not of interest in the current model (i.e., utter–listen for GLM_vi; view–imagery for GLM_ul). Motion‐ and physiology‐related regressors (e.g., WM/CSF signals) were not added because these sources of noise had already been removed during ICA denoising. All regressors were convolved with the canonical hemodynamic response function; in GLM_vi, time and dispersion derivatives were also included. A 128‐s high‐pass filter and the FAST autoregressive model were applied.

#### 2.6.2 Second Level GLM Model and Analysis

Regarding the second‐level analysis, we focus on two contrasts of each role (sender and receiver) from a 2 × 2 × 2 full factorial design of each model (face map: face > control, vivid face map: (face5 hints (f5) > face2 hints (f2)) ∩ (f5—control5 hints(c5))). The statistical threshold was set at *p* < 0.001 with uncorrected at peak level and *p* < 0.05 with a family‐wise error (FWE) correction at the cluster level for the whole brain (Friston et al. 1996).

#### 2.6.3 Brain‐Behavior Relationships

We conducted group level multiple regressions to examine the relationship between face information‐related activation and behavioral performance and individual differences. The activation map of the imagery period in the receiver was set as the dependent variable, and the correct rate of the 4AFC, the visual dominance of the VVQ, and the face imagery score of VVIQ in the receiver were set as independent variables. All covariates were mean centered. Intercepts were included in the models.

### 2.7 Effective Connectivity

Dynamic causal modeling (DCM 12.5) was used to estimate effective connectivity (Friston et al. 2003; Zeidman, Jafarian, Corbin, et al. 2019; Zeidman, Jafarian, Seghier, et al. 2019). Our analysis focused on comparing connectivity during the utterance period (sender) and the imagery period (receiver). Utterance involves transforming visual face information into verbal output, whereas imagery involves reconstructing visual representations from auditory input. We examined how these two processes differ and overlap in their connectivity profiles and whether two conditions (f2 vs. f5) modulated connection strength differently.

#### 2.7.1 VOI Definition and Time Series Extraction

The goal of the DCM analysis was to compare connectivity patterns between the sender, who verbalized visual face information, and the receiver, who converted linguistic descriptions into mental face imagery. ROI selection was therefore grounded in this research question. Prior studies of face‐related visual imagery have identified a core network involving the left inferior frontal gyrus (lIFG), left intraparietal sulcus (lIPS), and left fusiform face area (lFFA) (Dijkstra et al. 2017, 2019; Spagna et al. 2021). To enable direct comparison across roles, we defined these three regions as ROIs for both the utterance and imagery DCMs. Our brain activation results supported this theory‐driven approach, in that all three ROIs showed robust, overlapping activation in both conditions, confirming that the same network was engaged across roles and thus provided a common functional space for comparison. This strategy aligns with standard DCM methodology (e.g., Zeidman, Jafarian, Corbin, et al. 2019), where analysis is based on regions reliably activated by experimental manipulation.

Group‐level peak coordinates for each ROI were extracted from the face‐contrast maps for the utterance and imagery periods. The imagery‐IPS peak was defined using the face > 0 contrast because this region reached significance only in the vivid‐face map. To accommodate inter‐individual variability, subject‐specific peaks were then identified. For each participant, a 12‐mm sphere was centered on the group‐level peak and intersected with the corresponding anatomical mask (lIFG, lIPS, or lFuG) from the JuBrain Atlas (Eickhoff et al. 2005, 2006, 2007). The subject‐specific peak was defined as the nearest local maximum within this constrained search volume.

To prepare the BOLD time series for DCM, we first generated two new GLMs based on the original first‐level models. In these GLMs, the main task effects (the view period in GLM_vi and the listen period in GLM_ul) were re‐modeled as nuisance regressors to isolate trial‐level variance. Time series from the six runs were concatenated, and the first eigenvariate was extracted from all significant voxels (*p* < 0.05, uncorrected) within a 6‐mm sphere centered on the subject‐specific peak. These voxels were defined using the face > 0 contrast, following recommendations by Zeidman, Jafarian, Corbin, et al. (2019). Six participants who showed no significant activity in at least one ROI during imagery were excluded from further analysis.

#### 2.7.2 First Level DCM Model Specification

First‐level models were constructed as fully connected, single‐state, bilinear, and mean‐centered, following Zeidman, Jafarian, Corbin, et al. (2019). The models included intrinsic connections and self‐connections (matrix A), modulatory effects of the low‐ and high‐information conditions (f2 and f5; matrix B) and driving inputs from all face stimuli (matrix C). We modeled f2 and f5 as separate modulatory inputs rather than a single parametric regressor because our primary interest was in characterizing the connectivity patterns associated with each information condition independently, rather than assuming a monotonic relationship between information amount and connectivity strength. This approach allowed us to examine whether sparse and rich information conditions engage distinct modulatory dynamics within each communicative role and enabled flexible contrasts—both between information conditions within each role (e.g., f5 vs. f2 for visualization), and between roles within each information condition (e.g., visualization vs. verbalization under f2). Given our interest in comparing network dynamics during verbalization and mental visualization, we designated lIFG and lFFA as driving input regions for both the utterance and imagery models, based on prior literature (Lee et al. 2022; Dijkstra et al. 2017, 2019; Spagna et al. 2021).

#### 2.7.3 Second Level PEB Analysis

After estimating first‐level models, we applied Parametric Empirical Bayes (PEB) to incorporate individual parameter estimates into a group‐level model. PEB considers both the magnitude and uncertainty of each parameter, providing an estimate of the average effect across participants (Friston et al. 2016). Here, we focused on the group effects for each DCM parameter without the addition of other covariates. To explore the effective connectivity architecture, we used automatic model search implemented in the DCM‐PEB framework. This employs Bayesian Model Reduction (BMR) to conduct an exhaustive search across the entire model space. This employs Bayesian Model Reduction (BMR) to conduct an exhaustive search across the entire model space. The fully connected model described above served as the parent model (search space). Starting from this parent model, BMR automatically pruned parameters and evaluated the model evidence (Free Energy) of each reduced topology relative to the parent (Zeidman, Jafarian, Seghier, et al. 2019; Kuhnke et al. 2021). This process is mathematically equivalent to testing a full set of nested causal models, identifying a subset of plausible models (Occam's window) that best explain the data. Bayesian Model Averaging (BMA) was applied to obtain a robust estimate of effective connectivity that explicitly accounts for the uncertainty across these competing topologies. The BMA procedure aggregates the estimates from the identified plausible models, weighting them by their respective log‐model evidence. Consequently, the reported connectivity parameters represent a consensus across the most probable model structures rather than a selection of a single model. Only parameters with posterior probability > 0.95 after BMA were considered significant. To compare modulatory effects between the f2 and f5 conditions, we derived the posterior distribution of their difference for each connection using the group‐level posterior mean and full covariance matrix obtained from BMA. This procedure preserves both posterior uncertainty and covariance between parameters. A posterior probability greater than 0.95 was interpreted as strong evidence that modulatory effects differed between conditions. In addition, we compared the summed forward (lFFA → lIFG + lFFA → lIPS) and backward (lIFG → lFFA + lIPS → lFFA) connectivity strengths associated with lFFA across conditions using the same Bayesian contrast approach.

To directly compare effective connectivity between the two communicative roles, we performed a third‐level PEB analysis. First, a second‐level PEB model was constructed for each participant based on the contrast between the utter and imagery GCMs. The third‐level PEB model then estimated the group average of these differences, thereby identifying connections that were reliably distinct between the two communicative roles. Parameters with a posterior probability exceeding 0.95 were considered to show strong evidence for a role‐related difference.

To complement the exploratory automatic model search, we conducted a hypothesis‐driven model comparison using nine predefined competing models to formally test our directional predictions regarding the dominant direction of information flow for each communicative role. The automatic model search and hypothesis‐driven model comparison parallel inference strategies within the PEB framework that operate over different model spaces: the former evaluates all possible reduced models from the full model, while the latter tests a constrained set of explicitly specified architectures. Full details of the model space definition and the corresponding results are provided in the Supporting Information (Figures S5–S6).

## 3 Results

### 3.1 Behavioral Performance

**FIGURE 2:**
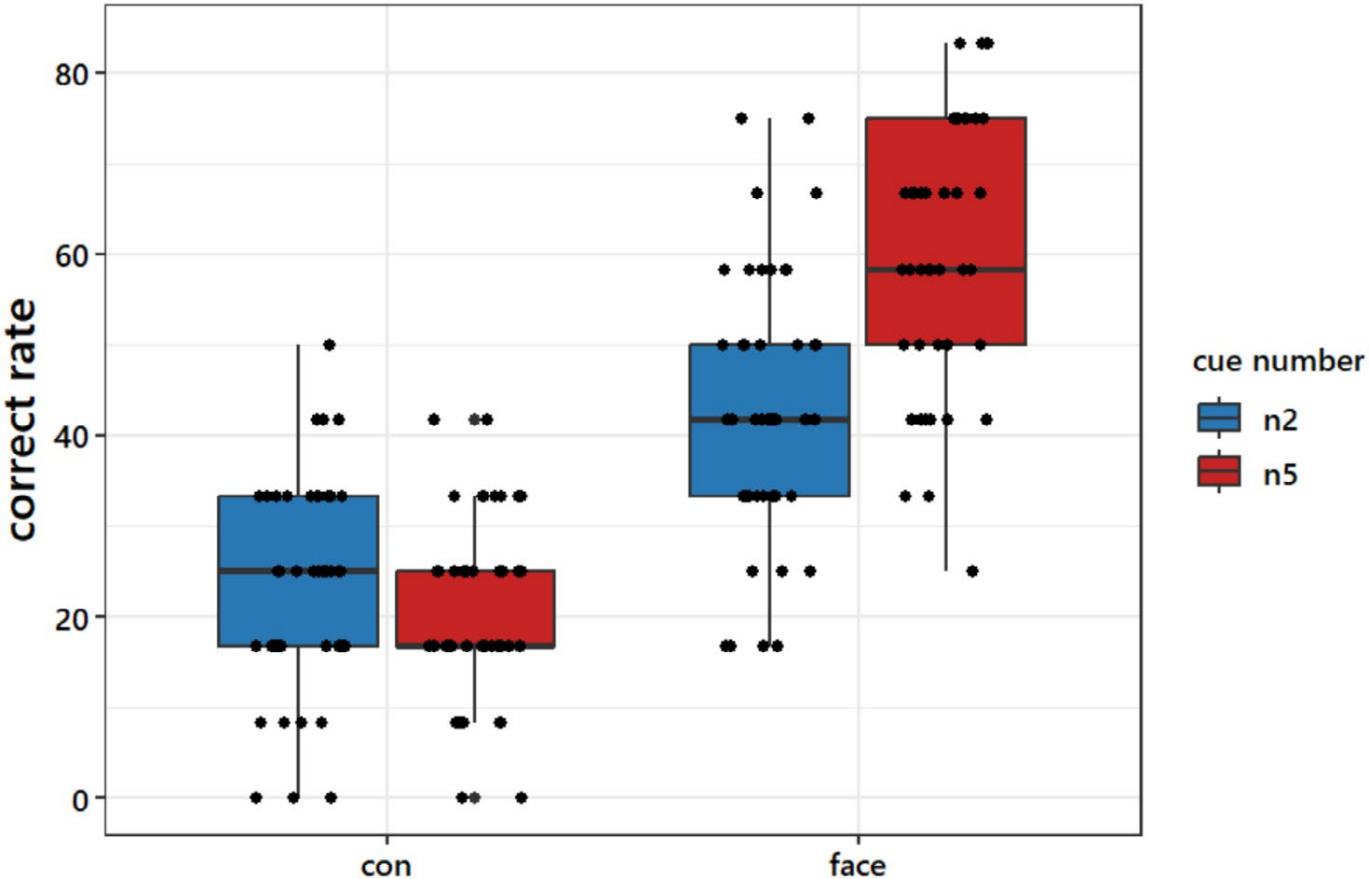
Behavioral performance. Correct rate of the receiver in a 4AFC task in the control and face conditions. The left panel shows performance under the control condition, while the right panel presents performance under the face condition. In both panels, the blue bars represent accuracy for the 2‐cue condition, and the red bars represent accuracy for the 5‐cue condition. The *y*‐axis represents accuracy as a percentage.

We examined the effects of category information and information amount on participants' performance using a 2 × 2 factorial analysis of variance (ANOVA) design. Results revealed significant main effects for category of information (*F*(1,45) = 215.97, *p* < 0.001, $\eta^{2}$= 0.83) and the information amount (*F*(1,45) = 15.70, *p* < 0.001, $\eta^{2}$= 0.26), as well as a significant interaction between them (*F*(1,45) = 22.00, *p* < 0.001, $\eta^{2}$ = 0.33). Post hoc *t*‐tests showed that participants' accuracy was significantly higher when making choices based on f5 than when making choices based on f2 (f5: mean (*M*) = 59.78, standard deviation (SD) = 14.94; f2: *M* = 43.48, SD = 14.79; *t*(45) = 5.36, *p* < 0.001; Figure 2). However, no statistically significant difference in accuracy was found between c5 and c2 (c5: *M* = 22.28, SD = 11.66; c2: *M* = 24.09, SD = 11.82; Figure 2).

### 3.2 fMRI Results: Brain Activation

#### 3.2.1 Shared and Distinct Activations for Verbalization and Visualization

**FIGURE 3:**
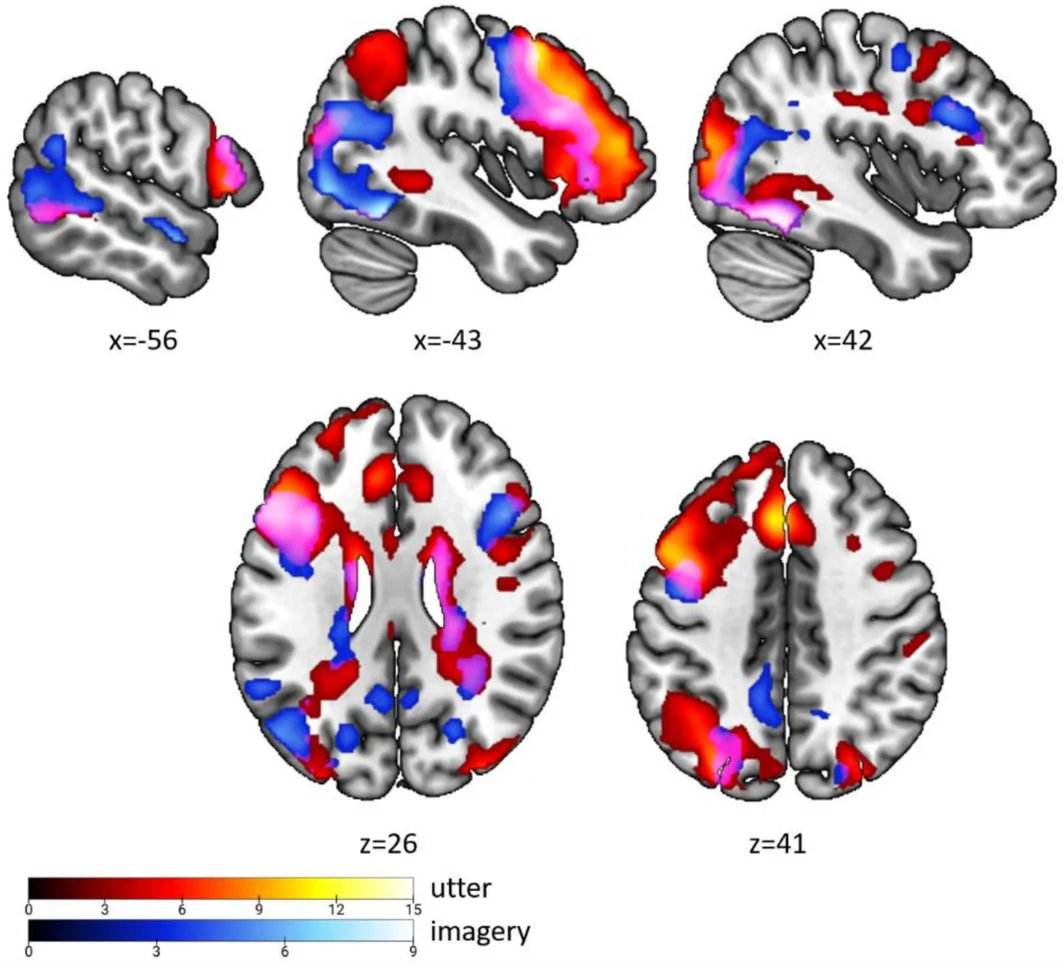
Whole brain activation map of face effect. Superimposition of the group activation map associated with the contrast utter_face > utter_control (in red) and the contrast imagery_face > imagery_control (in blue) from separate full factorial designs. Commonly activated brain areas are displayed in pink.

To characterize how verbalization and visualization differ and resemble one another in their neural implementation, we compared the activation maps for the two processes (Figure 3). This analysis revealed both a shared neural substrate and process‐specific activations. A core set of regions supporting visuo‐linguistic transformation was engaged by both tasks, including the bilateral fusiform gyrus (FuG), the left inferior frontal gyrus (IFG), the left inferior parietal area, and the left middle temporal gyrus (MTG) (Figure 3, pink overlay). Beyond this shared substrate, we identified distinct activations unique to each process. During verbalization in sender role, face‐related activation (Figure 3, red) was observed in the bilateral striate and extrastriate cortex (V1, V3, V4), bilateral pre‐SMA (extending into the left middle frontal gyrus), bilateral SPL and IPS, the paracingulate cortex (ParaCC), and bilateral thalamus and caudate nucleus (Table S1; Figure S1). In contrast, during mental visualization in receiver role, face‐related activation (Figure 3, blue) emerged in the left superior temporal gyrus (STG), left IPL/angular gyrus (AG), right amygdala, bilateral precuneus, and right IFG and right IPS (Table S3; Figure S3).

#### 3.2.2 Effect of Information Richness on Activation

**FIGURE 4:**
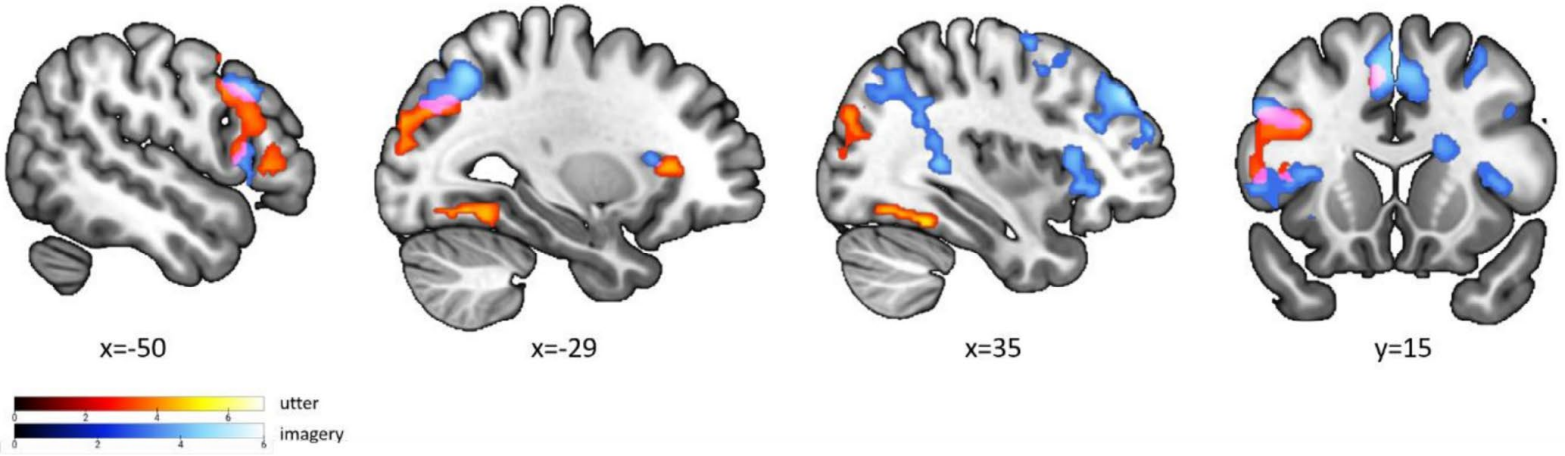
Whole brain activation map of vivid face effect. Superimposition of the group activation map associated with the contrast utter_(f5 > f2) ∩ utter_(f5 > c5) (in red) and the contrast imagery_(f5 > f2) ∩ imagery_(f5 > c5) (in blue) from the full factorial design of utter and imagery period. Commonly activated brain areas are displayed in pink.

We next examined how neural responses scale with the complexity of facial information by comparing f5 versus f2 within each role. This analysis revealed both convergent and divergent patterns of information‐dependent activation across encoding and decoding (Figure 4).

Convergent sensitivity to information richness appeared in a set of regions that showed greater activation for richer information across both tasks (Figure 4, pink). This shared network included the bilateral IPS (with broader anterior–posterior engagement during decoding and more focal activation during encoding), left IFG (extending into the anterior insula/MFG during encoding and into the insula during decoding), and left pre‐SMA (with ParaCC extension primarily during encoding).

We also observed clear role‐specific effects. During encoding (sender: (f5 > f2) ∩ (f5 > c5)), richer information additionally recruited the bilateral FuG extending into the occipital fusiform gyrus (OFuG), encompassing face‐selective regions in the FFA and OFA (Figure 4, red; Table S2). During decoding (receiver: f5 > f2), richer information uniquely engaged V1, bilateral SPL/precuneus, right MFG extending into the insula, and the bilateral caudate (Figure 4, blue; Table S4; Figure S4).

#### 3.2.3 Brain‐Behavior Interaction

**FIGURE 5:**
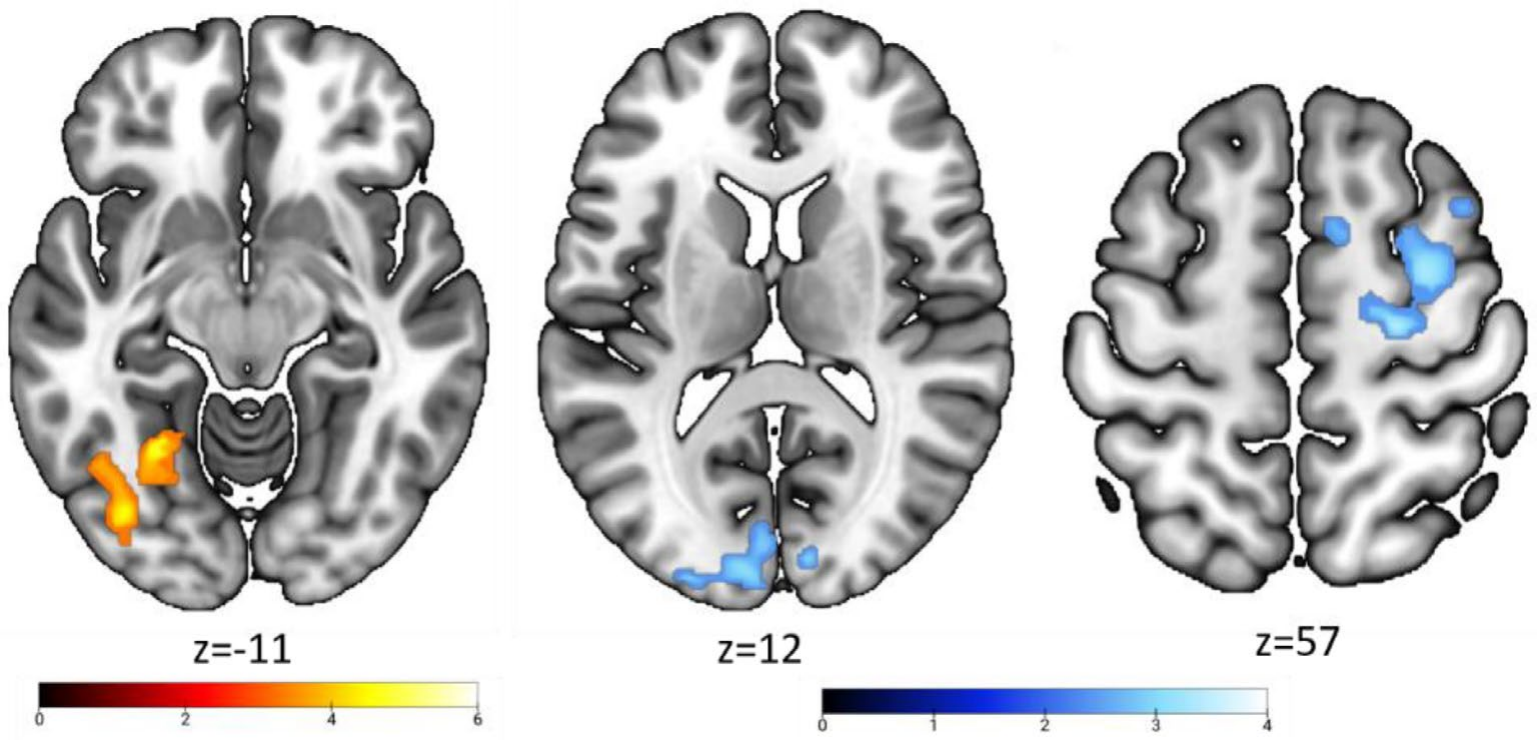
Relationship between neural activation, task performance, and visual‐verbal cognitive style. Positive correlation between imagery activation and task performance, with stronger activation linked to better performance (orange in left panel). Association between visual dominance and brain activation with increased facial information (light blue in middle and right panels).

We examined the relationship between face‐specific neural activation and individual performance on the task. We conducted multiple regression analyses using the main effect of face contrast (face > control) during imagery as the dependent variable and the difference in accuracy between the face and control conditions as the independent variable. The results showed a significant positive correlation between the level of activation in the left FuG (from temporo‐occipital to occipital region) and the individual's task performance (Figure 5 orange). This finding suggests that individuals with stronger face‐specific activation in the left FuG show better performance in processing face information compared to non‐face stimuli.

Furthermore, we examine whether individuals' cognitive style for embodied information is related to the imagine effect. To assess the relationship between the amount of facial information and individual visual‐verbal cognitive style, we conducted another multiple regression analysis. The difference in the amount of facial information presented in the experimental conditions (i.e., f5 > f2) was used as the dependent variable, and the individual's visual dominance score on the VVQ was used as the independent variable. The results showed that the activations in the primary visual cortex and the right MFG/SFG had a significant positive correlation with the individual's degree of visual dominance (Figure 5 light blue), although the statistical threshold was relatively lenient (*p* < 0.05 FWE correction at the cluster level under the uncorrected *p* < 0.005 at peak level). This result suggests that as the amount of facial information presented increases, individuals with a higher degree of visual dominance exhibit stronger activation in the primary visual cortex and the right prefrontal cortex.

### 3.3 fMRI Results: Effective Connectivity

The effective connectivity results reported below were obtained using automatic model search within the PEB framework, an exploratory procedure that identifies reliably modulated connections from a full model. A complementary hypothesis‐driven analysis is reported in Supporting Information Section S2 (Figures S5–S6).

#### 3.3.1 DCM Results of Uttering

**FIGURE 6:**
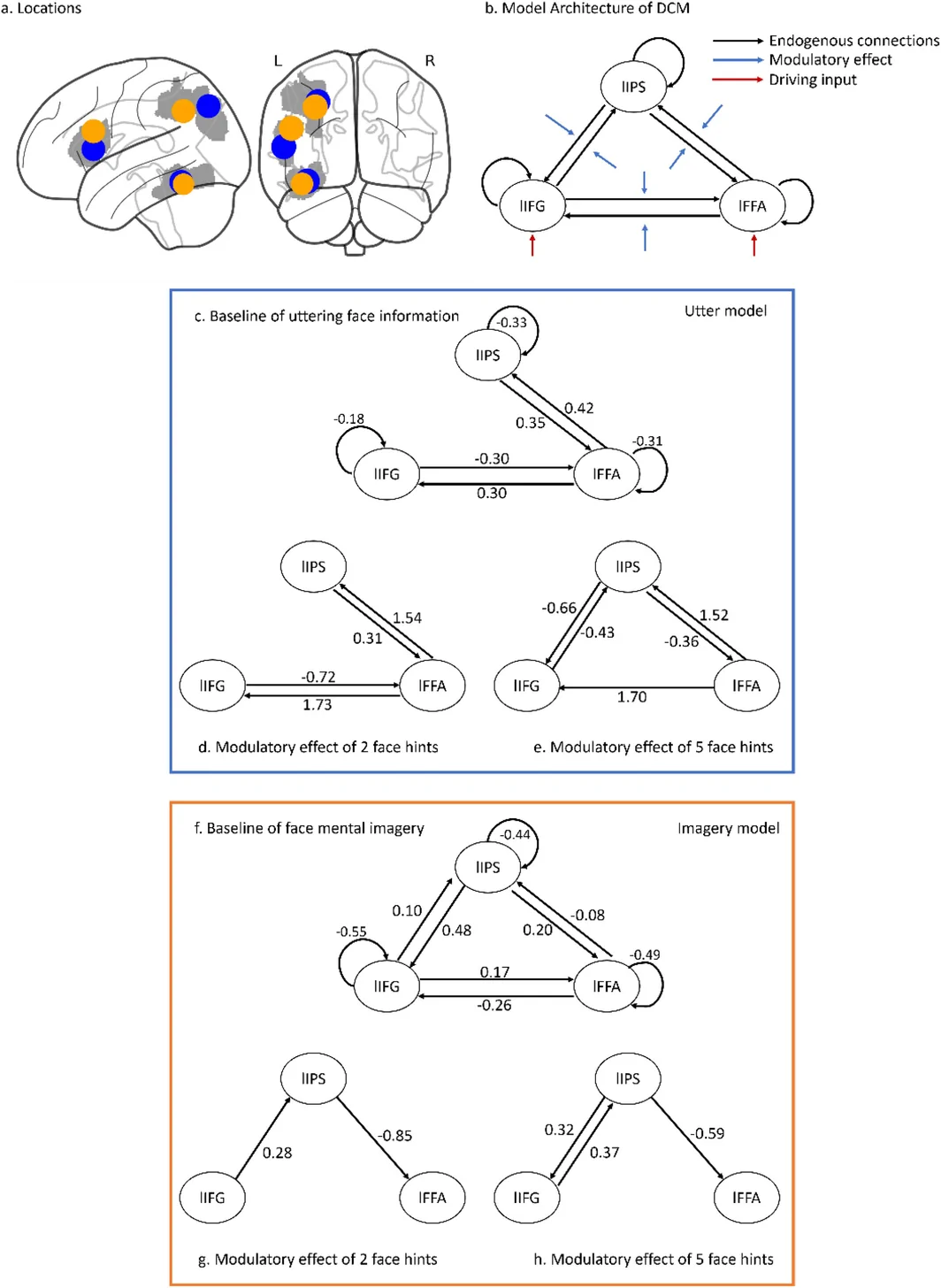
Utter‐ and imagery‐DCM model. (a) Locations of ROIs (spheres centered on group‐level peak coordinates; for details, see Section 2.7.1 for VOI definition and time series extraction; anatomy mask was showed in grey) (b) Architecture of DCM Model Specification. (c) PEB results of baseline connectivity for utter‐DCM. (d) PEB results of f2 modulation effect on utter‐DCM. (e) PEB results of f5 modulation effect on utter‐DCM. (f) PEB results of baseline connectivity for imagery‐DCM. (g) PEB results of f2 modulation effect on imagery‐DCM. (h) PEB results of f5 modulation effect on imagery‐DCM. Note that only suprathreshold parameters (P*p* > 0.95) are shown.

We performed a DCM‐PEB analysis focusing on uttering when participants were senders. The locations of the ROIs (spheres centered on group‐level peak coordinates) are shown in blue in Figure 6a, and the structure of the full DCM model design is illustrated in Figure 6b. The results for the A matrix and B matrices are presented in Figure 6c–e. The connection strengths of the parameters with a posterior probability greater than 0.95 (i.e., P*p* > 0.95) are reported. The A matrix represents the baseline connectivity between nodes. Reduced self‐inhibition was observed across three nodes. The lFFA transmits excitatory signals to both the lIPS and lIFG, while receiving inhibitory feedback from lIFG and excitatory feedback from lIPS (Fig. 6c). The B matrices illustrate how varying levels of facial information modulate effective connectivity between the modeled nodes. In the f2 condition (Fig. 6d), relative to baseline connectivity, the lIFG received robust excitatory feedback from lFFA while sending inhibitory signals back to lFFA. Concurrently, lIPS received substantial excitatory input from lFFA and returned excitatory feedback. In the f5 condition, relative to baseline connectivity, lFFA exerted robust positive influences on both lIPS and lIFG; lIPS sent inhibitory feedback to lFFA; and notably, lIPS and lIFG exchanged mutual inhibitory signals (Fig. 6e). Bayesian posterior inference revealed no strong evidence for condition differences in connection strength from lFFA to lIFG (posterior mean difference = 0.04, P*p* = 0.59) or from lFFA to lIPS (posterior mean difference = 0.03, P*p* = 0.56), nor in the summed forward and backward connectivity strengths of lFFA (forward: posterior mean difference = −0.06, P*p* = 0.59; backward: posterior mean difference = 0.05, P*p* = 0.60).

#### 3.3.2 DCM Results of Imagery

Subsequently, the DCM‐PEB results focus on mental imagery when participants assume the role of the receiver. Spherical ROIs centered on group‐level peak coordinates are shown in yellow in Figure 6a, while the full DCM model design remained identical to that illustrated in Figure 6b. In the A matrix (Figure 6f), reduced self‐inhibition was observed in three nodes, with all inter‐regional connections proving significant. The lFFA exerts inhibitory effects on both lIPS and lIFG, while lIFG and lIPS each send excitatory effects to the other two nodes. In the f2 condition, relative to baseline connectivity, lIFG transmits an excitatory signal to lIPS, while lIPS sends an inhibitory signal to lFFA (Figure 6g). The modulatory effects of these two connections in f5 were largely comparable to those of f2 (Figure 6h).

Bayesian posterior inference revealed no strong evidence for condition differences in modulatory connection strength from lIFG to lIPS (posterior mean difference = 0.09, P*p* = 0.87) or from lIPS to lFFA (posterior mean difference = 0.26, P*p* = 0.92), as neither exceeded the predefined criterion (P*p* > 0.95). Notably, f5 showed an additional excitatory effect from lIPS to lIFG that was not observed in f2. The summed forward and backward connectivity strengths of lFFA did not differ between the f2 and f5 conditions (forward: posterior mean difference = 0.11, P*p* = 0.83; backward: posterior mean difference = 0.27, P*p* = 0.92).

The modulatory connectivity results reported in Sections 3.3.1 and 3.3.2 were derived from an exploratory automatic model search within the PEB framework. To formally evaluate our directional predictions, we conducted a complementary hypothesis‐driven Bayesian model comparison across nine predefined architectures. This analysis converged with the exploratory findings, supporting top‐down modulatory connectivity for visualization and distributed, network‐wide modulation for verbalization (see Supporting Information Section S2 and Figures S5–S6 for details).

#### 3.3.3 Compare Between Two DCM‐PEB Models

**FIGURE 7:**
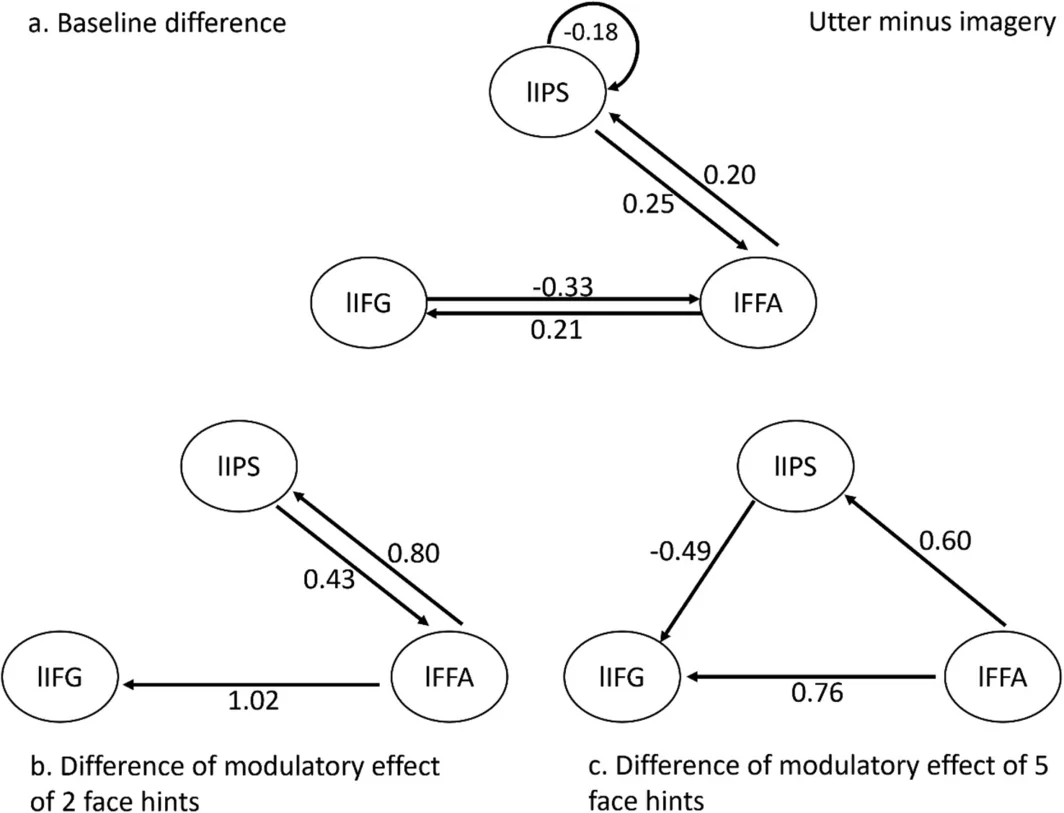
Results of third level PEB. Comparison of the utter‐DCM and imagery‐DCM models using the Bayesian contrast (GCM_utter > GCM_imagery). Only parameters with P*p* > 0.95 are shown. (a) Baseline connectivity difference between utter and imagery DCM (b) Difference of f2 modulatory connectivity between utter and imagery DCM. (c) Difference of f5 modulatory connectivity between utter and imagery DCM.

To investigate the differences in model space between the utter‐DCM model and the imagery‐DCM model, we performed a third‐level PEB analysis. Firstly, a second‐level PEB model was created for each individual using the contrast (GCM_utter > GCM_imagery). Then, the third‐level PEB model incorporated parameters for the group average of the difference between the utter and imagery GCM models. Third‐level PEB results were reported for parameter estimates with a posterior probability exceeding 0.95. In baseline connectivity, the utter‐DCM exhibited stronger reduced self‐inhibition in the lIPS and enhanced excitatory connectivity from lIPS to lFFA. Additionally, lFFA formed excitatory intrinsic connections to lIPS and lIFG, while demonstrating inhibitory connections from lIFG to lFFA and excitatory connections from lFFA to lIFG (Figure 7a). Notably, these lIFG‐lFFA connections displayed reversed polarity in the imagery‐DCM. Regarding modulatory effects, both f2 and f5 conditions revealed excitatory modulation from lFFA to both lIFG and lIPS connections in the utterance‐DCM (Figure 7b,c), demonstrating robust bottom‐up influences during verbalization. Conversely, the imagery‐DCM showed an inhibitory modulatory effect from lIPS to lFFA in the f2 condition (Figure 7b) and an excitatory modulatory effect from lIPS to lIFG in the f5 condition (Figure 7c), both exhibiting opposite polarity compared to the utter‐DCM.

## 4 Discussion

In the present study, we explored the neural mechanisms underlying the transformation of visual information into verbal descriptions and verbal information into mental imagery within an interpersonal communication framework. This study is one of the first to examine how the brain handles these transformations within the same role‐switching communicative situation, providing a unique view of the bidirectional processing of visual and verbal information. Participants alternated between the roles of sender (verbalizing visual cues) and receiver (forming mental images from verbal descriptions). This design allowed us to examine both encoding and decoding processes in the same individuals. We identified key regions involved in these transformations by using whole‐brain activation analysis and furthered this understanding by using DCM to examine how these regions interact, revealing how well‐established top‐down and bottom‐up connectivity patterns are maintained across task periods in this integrated communicative setting. These analyses provide new insights into the neural mechanisms that support the dynamic exchange between visual and verbal information and highlight the adaptability of the brain in sequential communication.

### 4.1 The Core Visuo‐Linguistic Interface: Shared Control and Complexity Scaling

A central finding is the recruitment of overlapping neural regions—including the inferior frontal gyrus (IFG), posterior middle temporal gyrus (pMTG), inferior parietal lobule/intraparietal sulcus (IPL/IPS), and fusiform gyrus (FuG)—during both verbalization and visualization. This convergent activation pattern suggests that these regions constitute a domain‐general interface supporting the bidirectional transformation between visual and linguistic representations. This is consistent with neural‐reuse accounts in which the brain flexibly deploys shared computational machinery across tasks with similar representational demands (Anderson 2010; Hodgson et al. 2024).

Importantly, activation in the IFG and IPS scaled with the number of facial features to be processed (f5 vs. f2), indicating sensitivity to representational complexity. This modulation likely reflects increased executive demands: senders must select and verbalize multiple semantic attributes, whereas receivers must integrate them into coherent internal models. Comparable scaling effects in the IFG and parietal cortex have been observed across semantic‐control tasks when retrieval or selection demands increase (Humphreys et al. 2022; Jackson 2021; Hodgson et al. 2024). The functional relevance of this network was further supported by brain–behavior correlations: left FFA activation predicted overall task accuracy across roles, consistent with its role in representing and discriminating facial information (Chen et al. 2023).

### 4.2 Perceptual Parsing Versus Constructive Assembly: Distinct Mechanisms

Beyond this shared substrate, the two task roles engaged dissociable downstream mechanisms. When individuals acted as senders, activity followed a perceptual‐parsing profile. Enhanced activation in V1 and ventral‐temporal regions reflects bottom‐up encoding of visual detail, consistent with the hierarchical gradient from low‐level features to category‐selective representations in ventral temporal cortex (Grill‐Spector et al. 2016). Under higher complexity, recruitment shifted toward FFA/OFA rather than additional V1 involvement, suggesting increased reliance on category‐level representations during demanding feature discrimination (Rotshtein et al. 2005). In contrast, when individuals acted as receivers, activity followed a constructive‐assembly profile. In contrast, receivers showed enhanced recruitment of IPS and precuneus. IPS supports spatial attention and integration of visual features (Sestieri et al. 2017), while precuneus is involved in generating internal visual representations during imagery tasks (Spagna et al. 2021; Cavanna and Trimble 2006). Together, these regions enable construction of visual images from verbal descriptions (Kosslyn et al. 2001).

Generative depth was modulated by both task and individual traits. Early visual cortex was not reliably engaged during visualization, but activation emerged under higher information load and in individuals with strong visual‐imagery dominance. This aligns with evidence that V1 involvement increases when fine‐grained reconstruction is required or when individuals generate vivid internal images (Dijkstra et al. 2017; Keogh and Pearson 2018; Fulford et al. 2018).

### 4.3 Mechanistic Reconfiguration of Intrinsic Connectivity

The effective connectivity analyses indicate that the sender and receiver roles reflect two coordinated configurations of a shared intrinsic architecture, expressed through complementary shifts in coupling polarity between ventral visual cortex and frontoparietal control regions.

During verbalization (i.e., sender), excitatory lFFA → lIFG/lIPS coupling was prominent, consistent with task‐dependent shifts in effective connectivity wherein visual information from face‐processing regions is routed preferentially toward regions supporting semantic–lexical retrieval (Heim et al. 2009; Richardson et al. 2011) and attentional control (Bitan et al. 2005; Schurz et al. 2014). Feedback signals diverged into two pathways: lIPS provided excitatory modulation of lFFA, consistent with attentional gain enhancement (Serences and Kastner 2014; Buschman and Kastner 2015) and empirical demonstrations of top‐down modulation from parietal cortex to visual regions (Moore and Armstrong 2003; Lauritzen et al. 2009), whereas lIFG exerted inhibitory influence, potentially reflecting top‐down suppression of perceptual processing during internally‐driven verbalization, consistent with recent evidence for prefrontal modulation of visual cortex activity (Osada et al. 2024; Konen and Kastner 2024). In the mental visualization condition (i.e., receiver), these directions were reversed. lIFG and lIPS exerted excitatory influences on lFFA, providing the primary evidence for a top‐down generative mechanism in which higher‐order frontal and parietal regions drive face‐selective activity during internally driven visualization (Dentico et al. 2014; O'Craven and Kanwisher 2000; Ishai et al. 2002). This interpretation aligns with imagery research showing that internally generated signals can reconstruct category‐selective representations in ventral visual cortex, including the FFA, through coordinated recruitment of prefrontal and parietal regions rather than sensory‐driven input (Dijkstra et al. 2017; Pearson 2019). Conversely, bottom‐up lFFA → lIFG/lIPS coupling became inhibitory, indicating that feedforward pathways were suppressed and thus did not contribute to representational formation under semantic‐based visualization.

Taken together, verbalization and visualization form a reciprocal pair: one configuration amplifies incoming sensory evidence and constrains feedback, while the other amplifies top‐down predictions and gates sensory drive, allowing the same network to alternate flexibly between perceptual encoding and internal reconstruction.

### 4.4 Load‐Dependent Reconfiguration of Parietal Control

The exploratory modulatory analysis, together with the third‐level PEB comparison, indicated a complexity‐dependent reconfiguration of effective connectivity within a single frontoparietal–ventral visual circuit. In this network, the intraparietal sulcus (IPS) differentially modulated face‐selective visual cortex as a function of communicative role. This pattern was further supported by the complementary hypothesis‐driven model comparison, which confirmed the predicted architectural distinction—predominantly top‐down connectivity for imagery and distributed modulation for uttering (see Supporting Information Section S2 and Figures S5–S6).

Under verbalization, increasing complexity shifted IPS → lFFA coupling from excitatory to inhibitory, while lFFA maintained excitatory influences on frontal and parietal regions. This pattern aligns with evidence that parietal cortex regulates visual information flow through gating mechanisms (Panichello and Buschman 2021), and that IPS plays a critical role in binding content to context during working memory, with activity that is sensitive to task demands (Cai et al. 2020; Fulvio et al. 2023). The observed inhibitory modulation may reflect IPS down‐regulating visual drive to allow sequential description of multiple facial features without exceeding the constraints of speech production. Under visualization, higher complexity strengthened frontoparietal coupling while IPS imposed inhibitory modulation on lFFA. This inhibitory pattern differs from prior DCM findings showing increased excitatory top‐down connectivity to early visual cortex with greater imagery vividness (Dijkstra et al. 2017). The divergence may reflect differences between early visual cortex (V1/V2) and high‐level face‐selective regions, or between vividness‐based and complexity‐based modulation. Nonetheless, the pattern is consistent with evidence that IPS modulates task‐evoked functional connectivity between parietal and ventral visual regions (Hwang et al. 2020). The contrasting patterns across conditions suggest complementary strategies: verbalization gates visual flow to manage output demands, whereas visualization modulates sensory input during internal construction. Thus, the two roles reflect paired adjustments within a unified IPS‐centered system: verbalization meets complexity by constraining visual input, whereas visualization meets complexity by reinforcing frontoparietal maintenance and suppressing visual‐cortical activity to support stable feature construction.

### 4.5 Limitations

A methodological consideration concerns the inferential status of the effective connectivity analysis. The primary connectivity results were obtained through automatic model search within the PEB framework, an exploratory procedure that identifies reliably modulated connections without constraining the solution to predefined architectures. Although we conducted a complementary hypothesis‐driven model comparison that yielded convergent conclusions at the architectural level (see Supporting Information Section S2 and Figures S5–S6), the pre‐specified models were specified post hoc rather than derived from a strongly constrained theoretical model established prior to data collection. For studies in which clear a priori predictions about network connectivity can be formulated, hypothesis‐driven model comparison grounded in a well‐established theoretical framework would provide stronger inferential leverage. Nonetheless, the convergence between the two approaches increases confidence in the directional patterns reported here.

Additionally, the f2 and f5 conditions represent graded levels of information load within the same task and were modeled as separate modulatory inputs in DCM to enable condition‐specific comparisons across cognitive roles. Although supplementary analyses indicated reasonable independence between the two sets of modulatory parameters (see Supporting Information Section S2 and Figure S7), we cannot entirely exclude the possibility that residual shared variance between these graded conditions slightly reduced sensitivity to detect connectivity differences between f2 and f5. Future studies could extend the present approach by using parametric modulation designs or experimental paradigms that more strongly dissociate levels of information load.

## 5 Conclusion

This study provides neural evidence for the bidirectional transformation of visual and verbal information in real‐time interpersonal communication. Using an introduction‐response paradigm with whole‐brain activation and DCM analyses, we identified common and distinct brain networks that flexibly adapt to verbalization and visualization demands. Our findings reveal dynamic interactions between sensory (visual–auditory) processing, language networks, and cognitive control, and provide a neural framework for understanding flexible multimodal communication.

## Author Contributions

**Yulei Shen:** conceptualization, paradigm design and programming, data acquisition, formal analysis, writing – original draft, visualization. **Takahiko Koike:** conceptualization, paradigm design, data acquisition, and writing – review and editing. **Shohei Tsuchimoto:** MRI data acquisition, technical support in data preprocessing, and writing – review and editing. **Ayumi Yoshioka:** MRI data acquisition and writing – review and editing. **Kanae Ogasawara:** MRI data acquisition and writing – review and editing. **Norihiro Sadato:** writing – review and editing, funding acquisition and supervision. **Hiroki C. Tanabe:** conceptualization, paradigm design, data acquisition, formal analysis, writing – original draft and review and editing, funding acquisition and project administration.

## Funding

This work was supported in part by a Grant‐in‐Aid for Scientific Research C #20K07721 (H.C.T.), #24K10481 (H.C.T.), A #24H00622 (N.S., T.K., H.C.T.), by JST SPRING JPMJSP2125 (Y.S.) from the Japan Society for the Promotion of Science, and by the Japan Agency for Medical Research and Development under Grant #JP18dm0107152 and #JP22dm0307005 (N.S.).

## Conflicts of Interest

The authors declare no conflicts of interest.

## Supporting information


**Figure S1:** Brain activation maps of face effect during verbalization displayed on inflated cortical surfaces. The left and right columns show the lateral, medial, and ventral views of the left and right hemispheres, respectively.
**Table S1:** Results of the whole‐brain univariate analysis for the face effect during verbalization. Activations were thresholded at a peak‐level threshold of *p* < 0.001 (uncorrected) with a cluster‐level familywise error (FWE) correction of *p* < 0.05. For each significant cluster, the cluster size (in voxels) and FWE‐corrected *p*‐value are reported, along with the peak‐level statistics (*T*‐value, uncorrected *p*‐value, and MNI coordinates) for local maxima. Cytoarchitectonic labels (probability > 25%) and macroanatomical labels were derived from the SPM Anatomy Toolbox.
**Figure S2:** Brain activation maps of vivid face effect during verbalization displayed on inflated cortical surfaces. The left and right columns show the lateral, medial, and ventral views of the left and right hemispheres, respectively.
**Table S2:** Results of the whole‐brain univariate analysis for the vivid face effect during verbalization.
**Figure S3:** Brain activation maps of face effect during visualization displayed on inflated cortical surfaces. The left and right columns show the lateral, medial, and ventral views of the left and right hemispheres, respectively.
**Table S3:** Results of the whole‐brain univariate analysis for the face effect during visualization.
**Figure S4:** Brain activation maps of vivid face effect during visualization displayed on inflated cortical surfaces. The left and right columns show the lateral, medial, and ventral views of the left and right hemispheres, respectively.
**Table S4:** Results of the whole‐brain univariate analysis for the vivid face effect during visualization.
**Figure S5:** Left: PEB‐BMC output of specified imagery model. Right: BMA of the parameters over survived imagery models.
**Figure S6:** Left: PEB‐BMC output of specified utter model. Right: BMA of the parameters over survived utter models.
**Figure S7:** Posterior parameter correlation matrix averaged across all 40 subjects. Left: Full correlation matrix, with rectangles highlighting regions of interest: green rectangles indicate correlations among B‐matrix parameters within the f2 condition; blue rectangles indicate correlations among B‐matrix parameters within the f5 condition; red rectangles indicate cross‐condition correlations between f2 and f5 B‐matrix parameters. Right: Enlarged view of the posterior correlation matrix restricted to B‐matrix parameters for f2 and f5.

## Data Availability

The data that support the findings of this study are available from the corresponding author upon reasonable request.
