# Supplementary material for: Neural Mechanisms of Bidirectional Visuo‐Linguistic Transformation in Interactive Communication
Source: Hum Brain Mapp. 2026 May 12;47(7):e70540. doi: 10.1002/hbm.70540 (PMC13161998; doi:10.1002/hbm.70540)
Supplement: Supplementary file 1 — Figure S1: Brain activation maps of face effect during verbalization displayed on inflated cortical surfaces. The left and right columns show the lateral, medial, and ventral views of the left and right hemispheres, respectively. Table S1: Results of the whole‐brain univariate analysis for the face effect during verbalization. Activations were thresholded at a peak‐level threshold of p < 0.001 (uncorrected) with a cluster‐level familywise error (FWE) correction of p < 0.05. For each significant cluster, the cluster size (in voxels) and FWE‐corrected p‐value are reported, along with the peak‐level statistics (T‐value, uncorrected p‐value, and MNI coordinates) for local maxima. Cytoarchitectonic labels (probability > 25%) and macroanatomical labels were derived from the SPM Anatomy Toolbox. Figure S2: Brain activation maps of vivid face effect during verbalization displayed on inflated cortical surfaces. The left and right columns show the lateral, medial, and ventral views of the left and right hemispheres, respectively. Table S2: Results of the whole‐brain univariate analysis for the vivid face effect during verbalization. Figure S3: Brain activation maps of face effect during visualization displayed on inflated cortical surfaces. The left and right columns show the lateral, medial, and ventral views of the left and right hemispheres, respectively. Table S3: Results of the whole‐brain univariate analysis for the face effect during visualization. Figure S4: Brain activation maps of vivid face effect during visualization displayed on inflated cortical surfaces. The left and right columns show the lateral, medial, and ventral views of the left and right hemispheres, respectively. Table S4: Results of the whole‐brain univariate analysis for the vivid face effect during visualization. Figure S5: Left: PEB‐BMC output of specified imagery model. Right: BMA of the parameters over survived imagery models. Figure S6: Left: PEB‐BMC output of specified utter model. Right: BMA o [file HBM-47-e70540-s001.docx]

Supplementary Materials

##
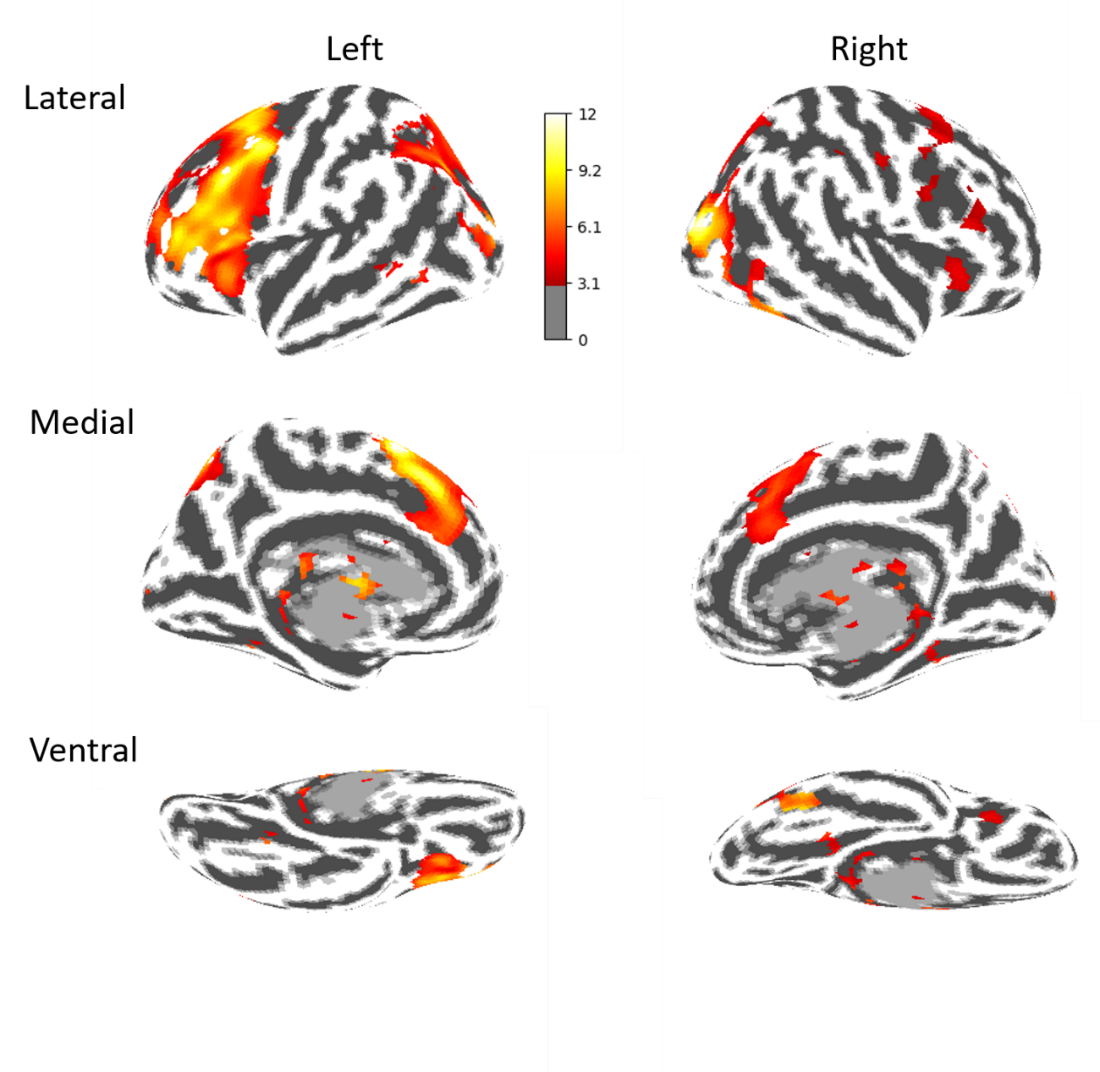
1. fMRI results: Brain activation

Fig. S1. Brain activation maps of face effect during verbalization displayed on inflated cortical surfaces. The left and right columns show the lateral, medial, and ventral views of the left and right hemispheres, respectively.

Table S1. Results of the whole-brain univariate analysis for the face effect during verbalization. Activations were thresholded at a peak-level threshold of p < .001 (uncorrected) with a cluster-level familywise error (FWE) correction of p < .05. For each significant cluster, the cluster size (in voxels) and FWE-corrected p-value are reported, along with the peak-level statistics (T-value, uncorrected p-value, and MNI coordinates) for local maxima. Cytoarchitectonic labels (probability > 25%) and macroanatomical labels were derived from the SPM Anatomy Toolbox.

| **Cluster level inference** | | **Peak level inference** | | **Hemisphere** | **MNI coordinates** | | | **Cytoarchitechture (Probability>25%)** | **Macroanatomy** |
| --- | --- | --- | --- | --- | --- | --- | --- | --- | --- |
| p (FWE-corr) | size | T | p (unc) |  | x | y | z |  |  |
| < 0.001 | 31149 | 14.15 | < 0.001 | R | 30 | -88 | 14 | hOc4d(V3A) | LOC_superior |
|  |  | 12.7 | < 0.001 | L | -4 | 14 | 66 | 6mr/preSMA | SFG |
|  |  | 12.51 | < 0.001 | R | -30 | 4 | 66 |  | MFG |
|  |  | 11.38 | < 0.001 | L | -4 | 20 | 48 |  | PCC |
|  |  | 11.22 | < 0.001 | R | 40 | -64 | -16 | FG2 | OFuG |
|  |  | 11.15 | < 0.001 | R | 44 | -52 | -20 | FG4 | FuG_TemOcc |
|  |  | 10.02 | < 0.001 | L | -50 | 20 | 2 | 44 | IFG_tri |
|  |  | 9.82 | < 0.001 | L | -10 | -74 | 56 | 7P(SPL) | LOC_superior |
|  |  | 9.61 | < 0.001 | R | 14 | -100 | 8 | V1 | OcciPole |


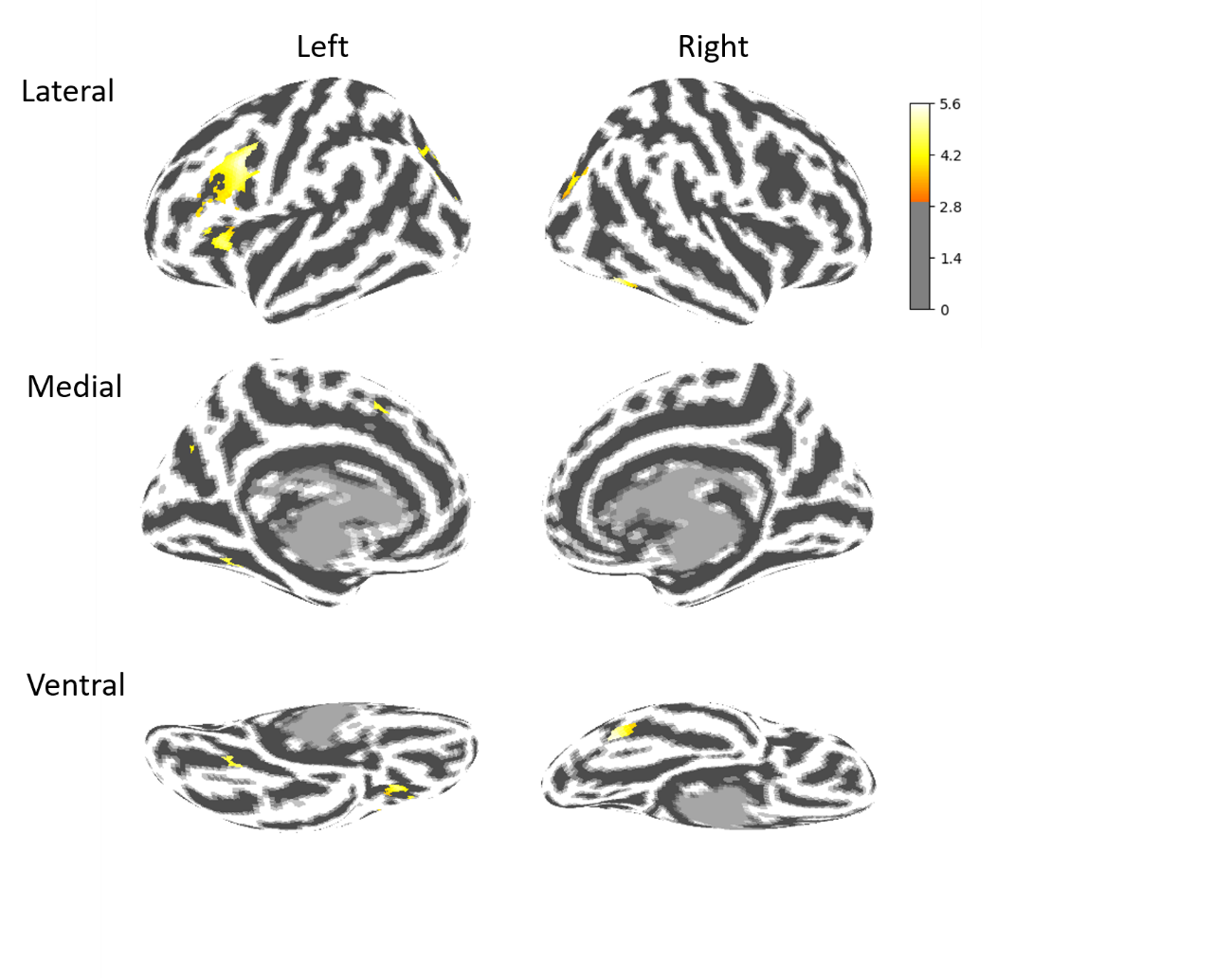


Fig. S2. Brain activation maps of vivid face effect during verbalization displayed on inflated cortical surfaces. The left and right columns show the lateral, medial, and ventral views of the left and right hemispheres, respectively.

Table S2. Results of the whole-brain univariate analysis for the vivid face effect during verbalization.

| **Cluster level inference** | | **Peak level inference** | | **Hemisphere** | **MNI coordinates** | | | **Cytoarchitechture (Probability > 25%)** | **Macroanatomy** |
| --- | --- | --- | --- | --- | --- | --- | --- | --- | --- |
| p (FWE-corr) | size | T | p (unc) |  | x | y | z |  |  |
| 0.003 | 369 | 6.32 | < 0.001 | R | 40 | -60 | -14 | FG2 | FuG_TemOcc |
|  |  | 5.32 | < 0.001 | R | 36 | -46 | -20 | FG4 |  |
|  |  | 4.78 | < 0.001 | R | 26 | -66 | -12 | hOc4v (V4(v)) | OfuG |
| 0.023 | 239 | 5.72 | < 0.001 | L | -36 | -52 | -16 | FG3 | FuG_TemOcc |
|  |  | 5.67 | < 0.001 | L | -38 | -62 | -16 | FG2 |  |
|  |  | 3.6 | < 0.001 | L | -26 | -76 | -14 | hOc4v (V4(v)) | OfuG |
| < 0.001 | 1390 | 5.22 | < 0.001 | L | -36 | 4 | 30 |  | PreCG |
|  |  | 4.77 | < 0.001 | L | -30 | 24 | 6 | Id7 | Insular |
|  |  | 4.53 | < 0.001 | L | -46 | 30 | 4 | OP9 | IFG_tri |
|  |  | 4.5 | < 0.001 | L | -44 | 6 | 32 | 44 | Mfg |
|  |  | 3.97 | < 0.001 | L | -50 | 18 | 24 | 44 | IFG_Oper |
| 0.023 | 237 | 4.6 | < 0.001 | R | 32 | -80 | 20 | hip4 (IPS) | LOC_superior |
|  |  | 4.47 | < 0.001 | R | 34 | -80 | 28 |  |  |
| 0.003 | 380 | 4.46 | < 0.001 | L | -30 | -72 | 30 | hip5 (IPS) | LOC_superior |
|  |  | 4.44 | < 0.001 | L | -30 | -86 | 26 | hip4 (IPS) |  |
|  |  | 4.06 | < 0.001 | L | -16 | -66 | 32 |  | Precuneous |
|  |  | 4.06 | < 0.001 | L | -28 | -86 | 16 |  |  |
| 0.046 | 195 | 4.41 | < 0.001 | L | -8 | 6 | 64 | 6mr/preSMA |  |
|  |  | 4.36 | < 0.001 | L | -6 | 12 | 48 | 6mr/preSMA | PCC |
|  |  | 3.94 | < 0.001 | L | 8 | 12 | 50 |  |  |


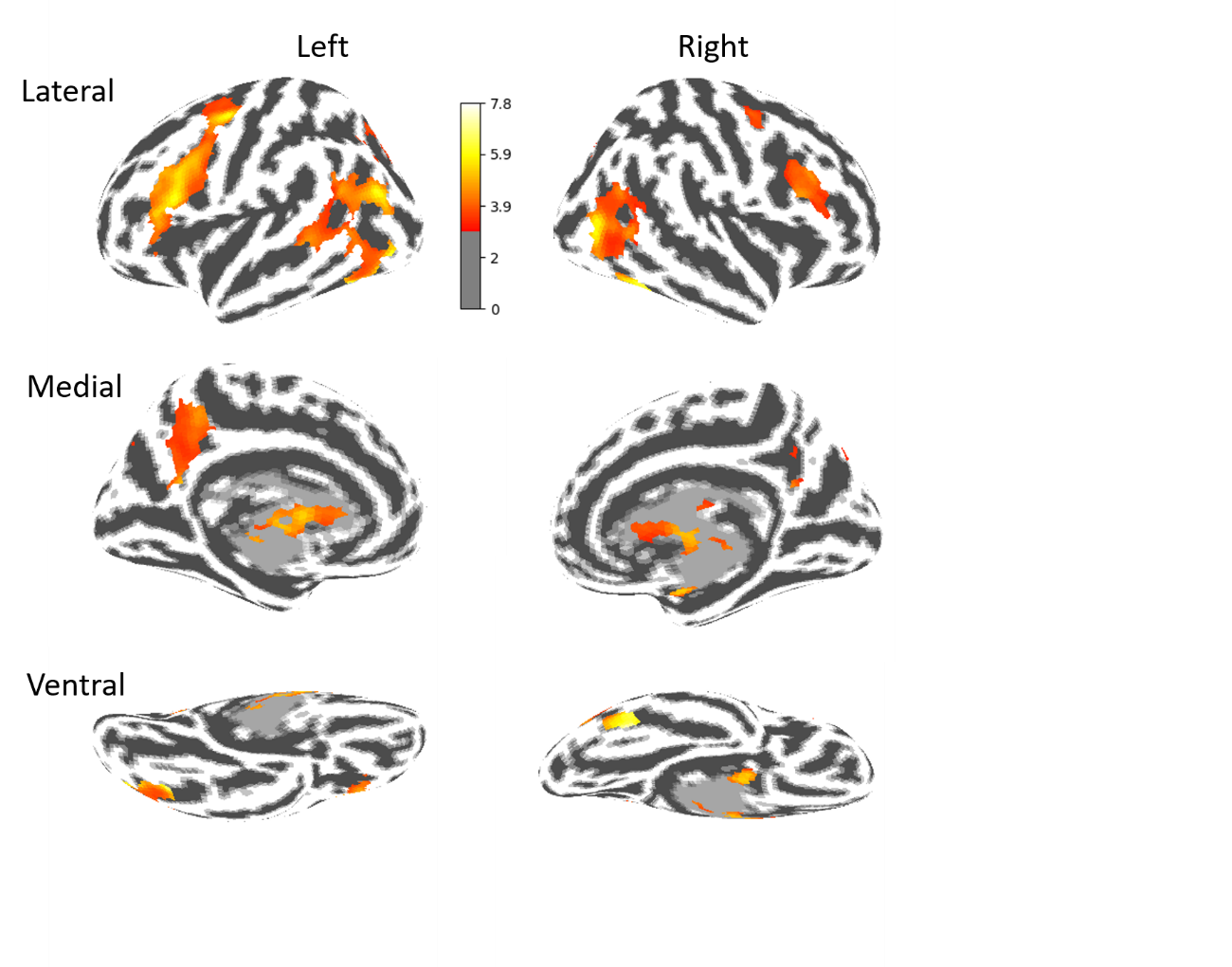


Fig. S3 Brain activation maps of face effect during visualization displayed on inflated cortical surfaces. The left and right columns show the lateral, medial, and ventral views of the left and right hemispheres, respectively.

Table S3 Results of the whole-brain univariate analysis for the face effect during visualization.

| **Cluster level inference** | | **Peak level inference** | | **Hemisphere** | **MNI coordinates** | | | **Cytoarchitechture (Probability > 25%)** | **Macroanatomy** |
| --- | --- | --- | --- | --- | --- | --- | --- | --- | --- |
| p (FWE-corr) | size | T | p (unc) |  | x | y | z |  |  |
| < 0.001 | 2986 | 9.22 | < 0.001 | L | -40 | -54 | -18 | FG4 | FuG_TeOc |
|  |  | 7.74 | < 0.001 | L | -52 | -4 | -16 | TE5 | STG_anterior |
|  |  | 6.95 | < 0.001 | L | -48 | -72 | 20 | PGp (IPL) | LOC_superior |
|  |  | 6.84 | < 0.001 | L | -42 | -78 | -8 | HOc4la | LOC_inferior |
|  |  | 4.96 | < 0.001 | L | -46 | -54 | 20 |  | AG |
|  |  | 4.74 | < 0.001 | L | -56 | -60 | 6 |  | MTG |
| < 0.001 | 6166 | 9.03 | < 0.001 | R | 44 | -52 | -18 | FG4 | FuG_TeOc |
|  |  | 7.01 | < 0.001 | R | 50 | -70 | 10 | hOc5 (VT/MT) | LOC_inferior |
|  |  | 6.94 | < 0.001 | R | 40 | -44 | -20 |  |  |
|  |  | 6.64 | < 0.001 | R | 2 | -6 | 4 |  | Thalamus |
|  |  | 5.09 | < 0.001 | R | 10 | -8 | 20 |  | caudate |
| 0.003 | 333 | 6.8 | < 0.001 | R | 20 | -8 | -14 | Amygdala (MF) | Amygadala |
|  |  | 4.61 | < 0.001 | R | 26 | -22 | -14 |  |  |
| < 0.001 | 2430 | 6.75 | < 0.001 | L | -38 | -6 | 46 |  | PreCG |
|  |  | 6.61 | < 0.001 | L | -48 | 18 | 24 |  | IFG_oper |
|  |  | 6.07 | < 0.001 | L | -54 | 24 | 20 | BA45 | IFG_tri |
|  |  | 6.02 | < 0.001 | L | -38 | 16 | 24 |  |  |
| < 0.001 | 1033 | 5.83 | < 0.001 | R | 8 | -54 | 20 |  | Precuneous |
|  |  | 5.69 | < 0.001 | L | -6 | -56 | 14 |  |  |
| < 0.001 | 486 | 5.55 | < 0.001 | R | 44 | 18 | 26 |  | IFG_oper |
|  |  | 4.4 | < 0.001 | R | 54 | 32 | 14 | BA45 | IFG_tri |
| 0.01 | 257 | 4.64 | < 0.001 | R | 34 | -6 | 48 |  | PreCG |
|  |  | 4.29 | < 0.001 | R | 44 | -4 | 48 |  |  |
| 0.012 | 246 | 4.54 | < 0.001 | R | 24 | -70 | 30 | hOP1(IPS) | LOC_superior |
|  |  | 3.8 | < 0.001 | R | 20 | -78 | 46 |  |  |


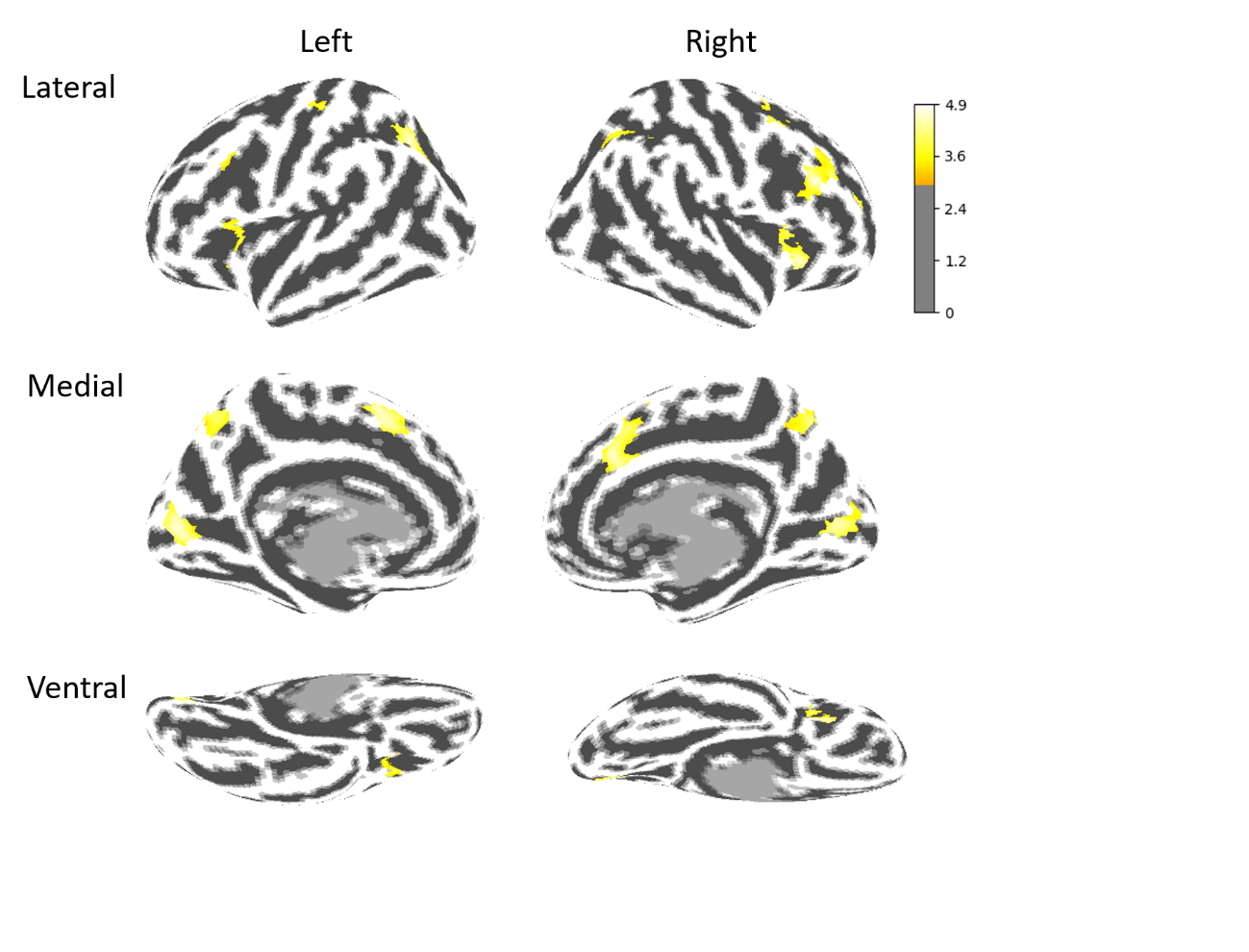


Fig. S4 Brain activation maps of vivid face effect during visualization displayed on inflated cortical surfaces. The left and right columns show the lateral, medial, and ventral views of the left and right hemispheres, respectively.

Table S4 Results of the whole-brain univariate analysis for the vivid face effect during visualization.

| **Cluster level inference** | | **Peak level inference** | | **Hemisphere** | **MNI coordinates** | | | **Cytoarchitechture (Probability > 25%)** | **Macroanatomy** |
| --- | --- | --- | --- | --- | --- | --- | --- | --- | --- |
| p (FWE-corr) | size | T | p (unc) |  | x | y | z |  |  |
| < 0.001 | 1296 | 5.63 | < 0.001 | R | 46 | 26 | 30 |  | MFG |
|  |  | 5.31 | < 0.001 | R | 40 | 36 | 38 |  | FP |
|  |  | 5.08 | < 0.001 | R | 32 | 24 | -2 | Id7 | Insular |
|  |  | 4.22 | < 0.001 | R | 36 | 20 | 10 | OP8 | FO |
|  |  | 3.47 | < 0.001 | R | 42 | 18 | 18 |  |  |
| 0 | 943 | 5.28 | < 0.001 | L | -2 | 10 | 58 | 6mr/preSMA | SFG |
|  |  | 5.06 | < 0.001 | R | 10 | 24 | 34 |  | PCC |
|  |  | 4.99 | < 0.001 | L | -4 | 14 | 48 | 6mr/preSMA | PCC |
|  |  | 4.56 | < 0.001 | R | 8 | 18 | 46 |  |  |
| < 0.001 | 467 | 5.01 | < 0.001 | L | -30 | -62 | 44 | hip3 (IPS) | LOC_superior |
|  |  | 3.58 | < 0.001 | L | -28 | -74 | 54 |  |  |
| < 0.001 | 913 | 4.93 | < 0.001 | L | -10 | -84 | 8 | hOc1 (v1) | Intracalcarine |
|  |  | 4.71 | < 0.001 | R | 12 | -78 | 12 |  |  |
| 0.001 | 371 | 4.59 | < 0.001 | R | 28 | 8 | 58 | 6d3 | MFG |
|  |  | 4.18 | < 0.001 | R | 34 | 0 | 62 |  |  |
| < 0.001 | 774 | 4.54 | < 0.001 | L | -8 | -66 | 54 | 7P (SPL) | Precuneous |
|  |  | 4.53 | < 0.001 | R | 8 | -64 | 48 |  |  |
|  |  | 3.48 | < 0.001 | R | 8 | -70 | 58 | 7A (SPL) | LOC_superior |
|  |  | 3.36 | < 0.001 | R | 12 | -74 | 48 |  |  |
| < 0.001 | 570 | 4.54 | < 0.001 | R | 36 | -40 | 8 |  |  |
|  |  | 4.28 | < 0.001 | R | 34 | -60 | 44 | hip6 (IPS) | LOC_superior |
|  |  | 4.16 | < 0.001 | R | 36 | -50 | 28 |  |  |
| 0.002 | 347 | 4.53 | < 0.001 | L | -12 | 2 | 16 |  | Caudate |
|  |  | 3.99 | < 0.001 | L | -18 | -4 | 24 |  |  |
| 0.008 | 270 | 4.4 | < 0.001 | L | -48 | 18 | 34 |  | MFG |
|  |  | 3.55 | < 0.001 | L | -36 | 2 | 34 |  |  |
|  |  | 3.19 | < 0.001 | L | -52 | 22 | 26 |  |  |
| 0.003 | 317 | 4.32 | < 0.001 | L | -44 | 20 | 4 | OP8 | FO |
|  |  | 4.11 | < 0.001 | L | -30 | 18 | 10 | Id6 | Insular |
|  |  | 3.97 | < 0.001 | L | -52 | 14 | 4 | BA44 | IFG_oper |
| 0.036 | 185 | 4.23 | < 0.001 | L | -38 | -28 | 52 | 4p | PostCG |
|  |  | 3.38 | < 0.001 | L | -40 | -34 | 62 | 3b |  |
| 0.001 | 403 | 4.13 | < 0.001 | R | 12 | 4 | 18 |  | Caudate |
|  |  | 4.12 | < 0.001 | R | 16 | 10 | 24 |  |  |

## 2. Hypothesis-driven Bayesian Model Comparison

**Model space definition**

We predicted that visualization would be most parsimoniously characterized by top-down connectivity from semantic control regions toward the visual cortex, whereas verbalization would engage more distributed connectivity due to its dual demands on visual analysis and semantic control. Nine competing models were defined to test these predictions.

Two constraints guided model construction. First, driving inputs entered through IFG and FG, but not IPS; therefore, IPS could only be activated via connections from IFG or FG. Second, the directionality of IFG–IPS interaction during visuo-linguistic transformation lacks clear prior empirical evidence. Rather than imposing unsupported assumptions, we incorporated IFG–IPS interactions within broader architectures—either as relay pathways in serial models or as bidirectional exchange in coordinated models.

**Top-down models:**

1. TD1 (Serial): IFG → IPS → FG
2. TD2 (Direct + Serial): IFG → FG, IFG → IPS → FG
3. TD3 (Coordinated): IFG → FG, IFG ⇄ IPS, IPS → FG

**Bottom-up models:**

1. BU1 (Direct): FG → IFG, FG → IPS
2. BU2 (Serial via IPS): FG → IPS → IFG
3. BU3 (Serial via IFG): FG → IFG → IPS
4. BU4 (Coordinated): FG → IFG, FG → IPS, IFG ⇄ IPS

**Reference models:** Full (all connections modulated); Null (no connections modulated).

**Results**

Visualization (Figure S5). TD1 (IFG → IPS → FG) was decisively favored (Pp = 0.84), outperforming all bottom-up models as well as the full model. The three top-down models collectively accounted for nearly all model evidence (cumulative Pp ≈ 1.00). These results support our prediction that visualization is driven by top-down connectivity and further specify a serial relay architecture from IFG through IPS to FG. The preference for TD1 over the full model indicates that this parsimonious top-down architecture explains the data better than a model in which all connections are modulated.

Verbalization (Figure S6). No directional model—neither bottom-up nor top-down—received support over the full model (Pp > 0.999). This finding indicates that the connectivity architecture underlying verbalization cannot be reduced to a single directional pathway. Instead, verbalization appears to require concurrent modulation across the entire network, consistent with its dual demands on visual analysis, semantic control, and inter-regional coordination within the SCN.

**Consistency between the two approaches**

For visualization, the automatic model search and the hypothesis-driven model comparison yielded highly consistent parameter estimates. For verbalization, the overall pattern converged, with minor differences at the parameter level: the hypothesis-driven comparison indicated additional IPS-to-IFG inhibitory modulation, a small IFG-to-IPS excitatory modulation, and stronger IFG-to-FG excitatory modulation that were not evident in the automatic model search. These differences likely reflect the distinct search strategies of the two approaches. The hypothesis-driven comparison selected the full model as the sole model within Occam’s window (Pp > 0.999), such that its Bayesian model averaging (BMA) is effectively equivalent to the unreduced full-model parameters. In contrast, the automatic model search, starting from the same full model, further pruned parameters with insufficient evidence and averaged across the resulting reduced models. This additional pruning explains the observed parameter-level differences and does not alter the core conclusions.


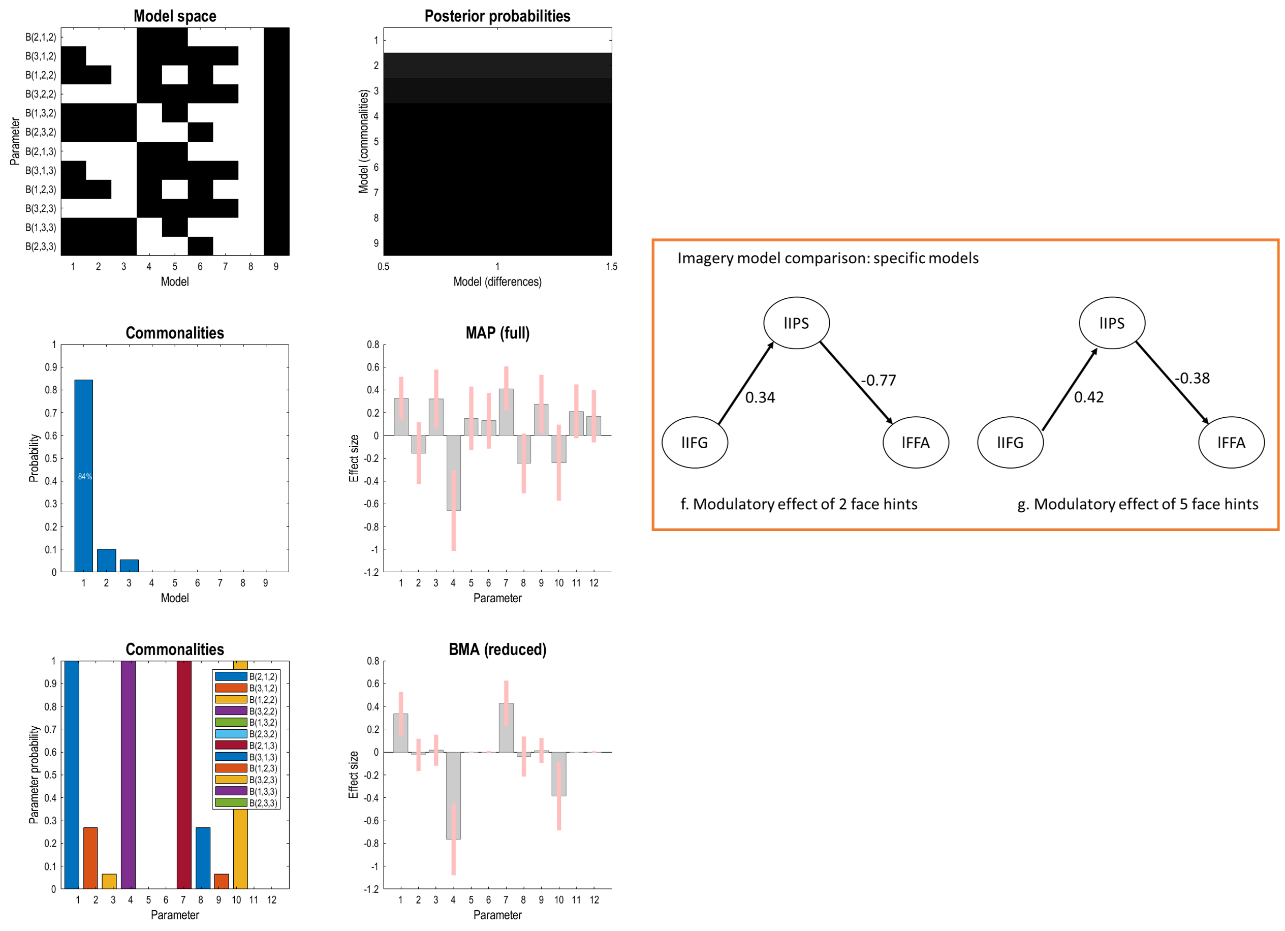


Fig. S5. Left: PEB-BMC output of specified imagery model. Right: BMA of the parameters over survived imagery models.

##
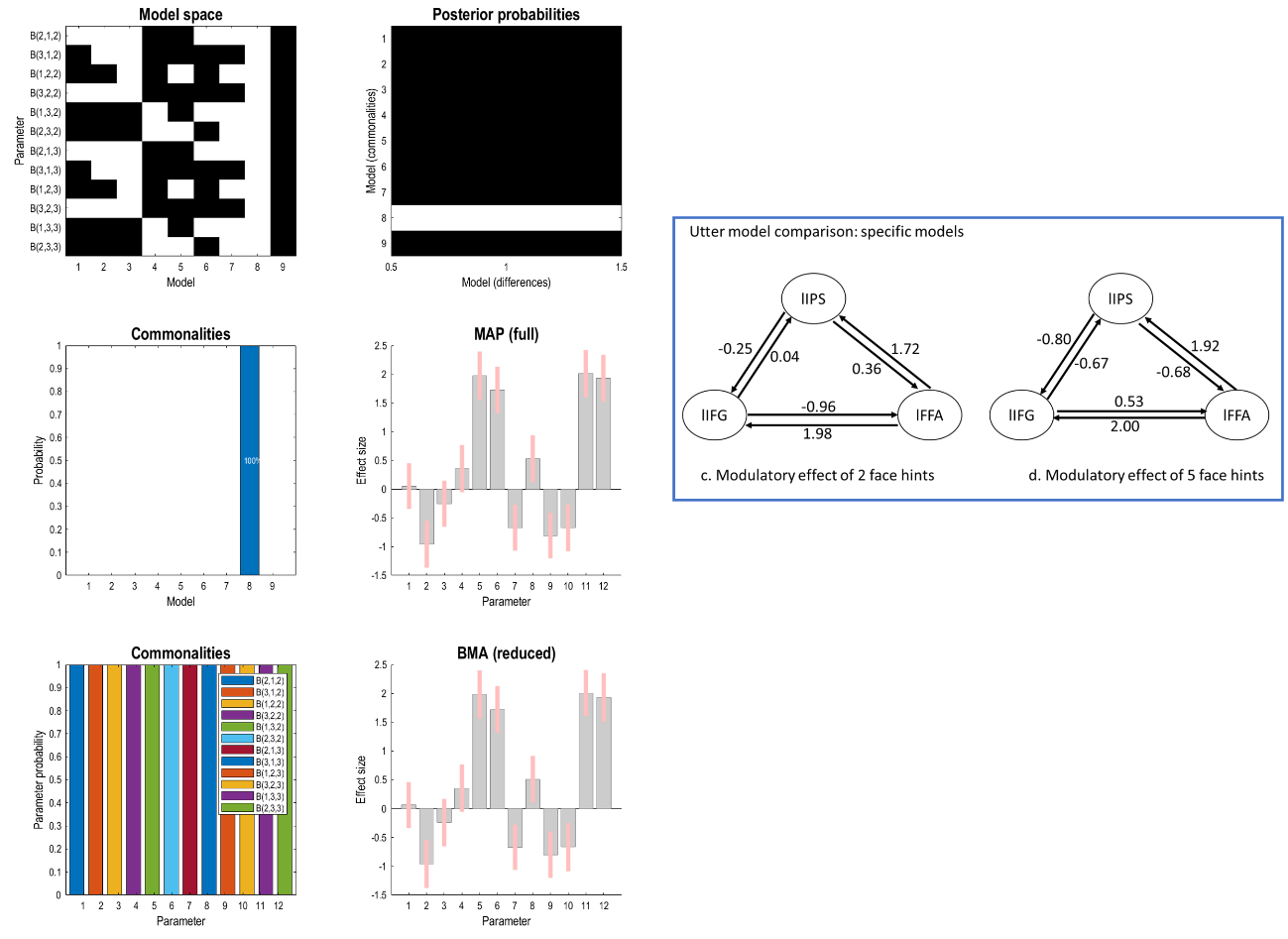
3. Assessment of Posterior Parameter Independence Between f2 and f5 Modulatory Inputs

Fig. S6. Left: PEB-BMC output of specified utter model. Right: BMA of the parameters over survived utter models.

To assess whether modeling f2 and f5 as separate modulatory inputs introduced problematic interdependence in the estimated B-matrix parameters, we examined the posterior covariance matrices (DCM.Cp) for each participant. Following Stephan et al. (2007), each participant’s posterior covariance matrix was normalized to obtain a conditional correlation matrix, which quantifies the degree to which parameter estimates covary given the data and priors. Group-mean posterior correlation matrices were then computed separately for the verbalization and visualization models.

The utterance model showed low posterior correlations between the f2- and f5-modulated B-matrix parameters (mean |r| = 0.08; range: −0.13 to 0.36; Figure S7, upper panels). The visualization model yielded similarly low values (mean |r| = 0.07; range: −0.08 to 0.46; Figure S7, lower panels). The only moderately elevated correlation (r = 0.46) was observed for the IFG→IPS connection modulated by f2 and f5; all other parameter pairs exhibited near-zero correlations. These results indicate that the f2 and f5 modulatory parameters were estimated with a reasonable degree of independence in both models, suggesting that their separate modeling did not introduce problematic parameter interdependence.


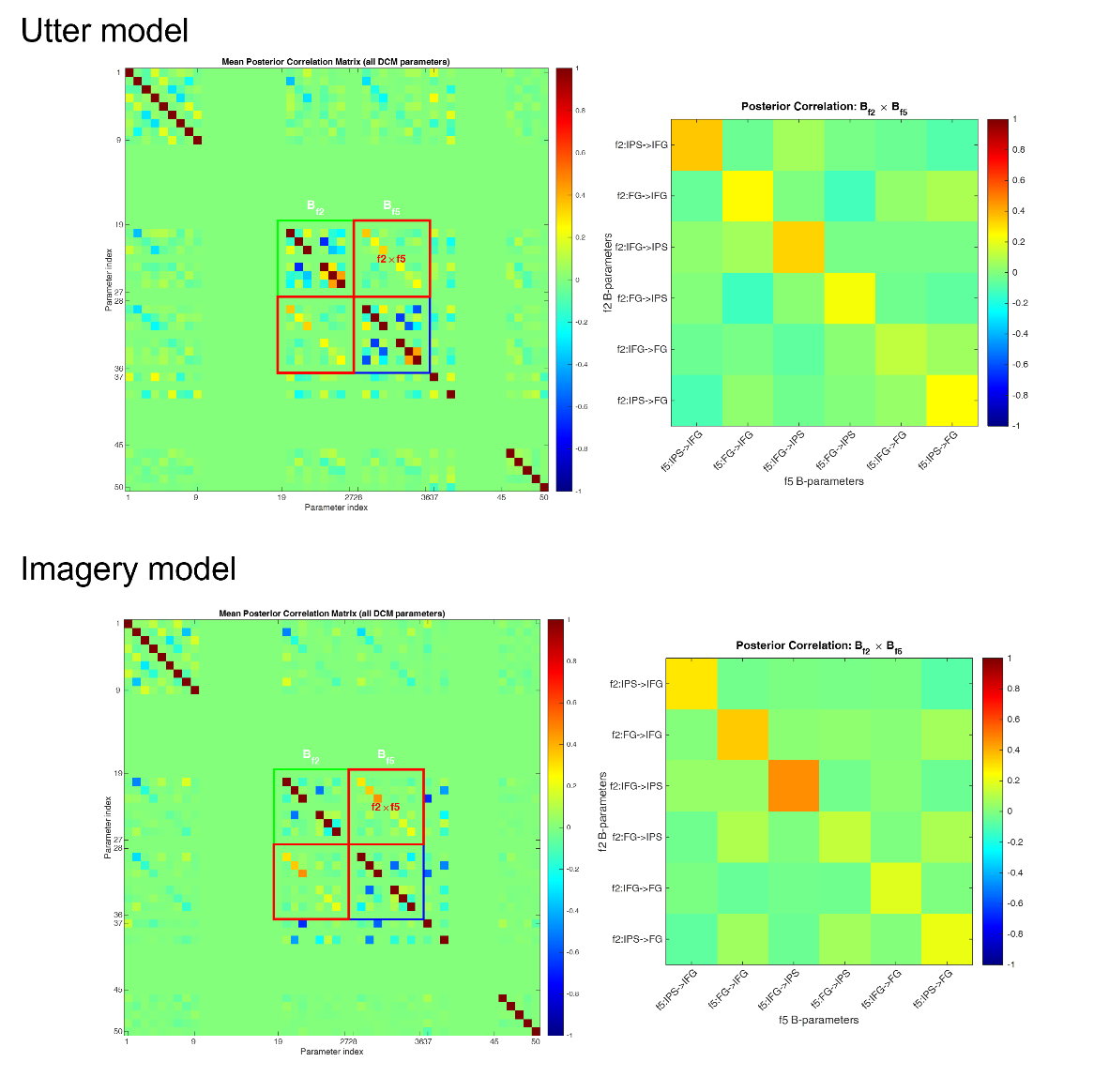


Fig. S7. Posterior parameter correlation matrix averaged across all 40 subjects. Left: Full correlation matrix, with rectangles highlighting regions of interest: green rectangles indicate correlations among B-matrix parameters within the f2 condition; blue rectangles indicate correlations among B-matrix parameters within the f5 condition; red rectangles indicate cross-condition correlations between f2 and f5 B-matrix parameters. Right: Enlarged view of the posterior correlation matrix restricted to B-matrix parameters for f2 and f5.
